# Sniffer mice discriminate urine odours of patients with bladder cancer: A proof-of-principle study for non-invasive diagnosis of cancer-induced odours

**DOI:** 10.1038/s41598-017-15355-z

**Published:** 2017-11-07

**Authors:** Takaaki Sato, Yoji Katsuoka, Kimihiko Yoneda, Mitsuo Nonomura, Shinya Uchimoto, Reiko Kobayakawa, Ko Kobayakawa, Yoichi Mizutani

**Affiliations:** 1 Biomedical Research Institute, National Institute of Advanced Industrial Science and Technology, Osaka, 563-8577 Japan; 2 Department of Urology, Portisland Hospital, Hyogo, 650-0046 Japan; 3 Department of Urology, Kameoka-Shimizu Hospital, Kyoto, 621-0834 Japan; 4 Department of Urology, Kyoto-Katsura Hospital, Kyoto, 615-8256 Japan; 5 Department of Urology, Nozaki Tokushukai Hospital, Osaka, 574-0074 Japan; 6 Institute of Biomedical Science, Kansai Medical University, Hirakata, Osaka, 573-1010 Japan; 7 Department of Medical Engineering, Faculty of Health Science, Aino University, Osaka, 567-0012 Japan; 8 Present Address: Kumagaya General Hospital, Saitama, 360-8657 Japan

## Abstract

Similar to fingerprints, humans have unique, genetically determined body odours. In case of urine, the odour can change due to variations in diet as well as upon infection or tumour formation. We investigated the use of mice in a manner similar to “sniffer dogs” to detect changes in urine odour in patients with bladder cancer. We measured the odour discrimination thresholds of mice in a Y-maze, using urine mixtures from patients with bladder cancer (Stage I) and healthy volunteers (dietary variations) as well as occult blood- or antibiotic drug metabolite-modulated samples. Threshold difference indicated that intensities of urinary olfactory cues increase in the following order: dietary variation < bladder cancer < occult blood < antibiotic drug metabolites. After training with patient urine mixtures, sniffer mice discriminated between urine odours of pre- and post-transurethral resection in individual patients with bladder cancer in an equal-occult blood diluted condition below the detection level of dietary variations, achieving a success rate of 100% (11/11). Furthermore, genetic ablation of all dorsal olfactory receptors elevated the discrimination thresholds of mice by ≥ 10^5^-fold. The marked reduction in discrimination sensitivity indicates an essential role of the dorsal olfactory receptors in the recognition of urinary body odours in mice.

## Introduction

Currently, cystoscopy and transurethral biopsy, which are highly invasive techniques, are required for the definitive diagnosis of bladder cancer. By developing methods to detect changes in urinary metabolites and/or odours that occur in patients with early stage cancer, non-invasive alternatives for early disease diagnosis may be possible. Urinary or serum metabolic profiles are being increasingly investigated to determine biomarkers for cancer or other diseases. When urinary metabolic profiles were analysed using dynamic solid-phase microextraction (SPME) and gas chromatography-mass spectroscopy (GC-MS), a significant difference was observed between cancer patients and healthy volunteers^1^. However, urinary and serum SPME-GC-MS profiles demonstrate high inter-individual variation, requiring principal component analysis to discriminate among cancer types^1–3^ or recent myocardial injury^4^, for instance. This requirement indicates an inability to classify individual samples in an overlapping range between positive and negative groups. Thus, further methodological improvement is required before detection of urine metabolic changes by GC-MS can be used universally.

Recently, there has been an increasing trend in the utilisation of electronic-nose (e-nose) devices for the analysis of exhaled breadth or other samples^5–10^. Besides the absence of standardised e-nose diagnostic methods, due to their poor specification of individual volatiles, e-nose sensor arrays are not particularly suitable for identifying individual compounds present in complex gaseous mixtures such as in the human breath^5^. In addition, it remains unclear whether and how the e-nose arrays are sensitive to disease-related signals, and if they can separate these signals from background variations such as individual-unique body odours in exhaled breath, urine samples and their modifications by diet (nutrition), or other environmental influences, reported in animal models^11–14^. The trade-off between performance (sensitivity, specificity, and general-purpose) and cost, as well as between specificity (single or multiple diseases and their subtypes) and sensitivity should be considered in designs of diagnostic instrumental analyses, especially in elemental sensor specificities. It is necessary to identify target molecular biomarkers and their disorder ranges relative the degree of disease severity.

Animal behavioural assays may offer a more sensitive alternative for discriminating urine odour. For example, sniffer dogs have been investigated for their ability to detect cancer^15–17^. Similarly, “sniffer mice,” which are trained with an olfactory cue, have been shown to discriminate between genetically determined mouse urine odours in a Y-maze, even though the mice had large dietary variation that influenced urine odours^13,14^. This odour discriminating ability of mice for weaker olfactory cues may add an advantage over the above-mentioned GC-MS and e-nose analyses, which are influenced by dietary metabolites^14^. Mice are also > 10^8^-fold more sensitive than GC-MS for the detection and discrimination of similar enantiomeric odours^18^ and sensitively discriminate between urine odours of other mice with or without experimental tumours in a proof-of-principle study^19^.

Moreover, understanding the relative intensities of olfactory cues in urine samples for the murine olfactory system would be useful for screening experimental conditions and potential biomarkers for diagnostic assays. Transgenic mice also provide a powerful tool for understanding functional roles of olfactory receptors in odour discrimination. In fact, impaired odour discrimination abilities in mice with genetically ablated dorsal olfactory receptors (ΔD mice) have revealed that these dorsal receptors are essential for recognising important olfactory cues such as predators^20^ and for supersensitive enantiomeric odour discrimination^18^. Therefore, we used sniffer mice to detect changes in urine odour in bladder cancer patients in a Y-maze. Thus, we also investigated reductions in urine odour discrimination sensitivity of ΔD mice, assessing the role of dorsal olfactory receptors for recognition of body odours.

## Results

### Odour discrimination threshold of mice for bladder cancer-induced urine odour change in equi-occult blood dilution series

**Figure 1:**
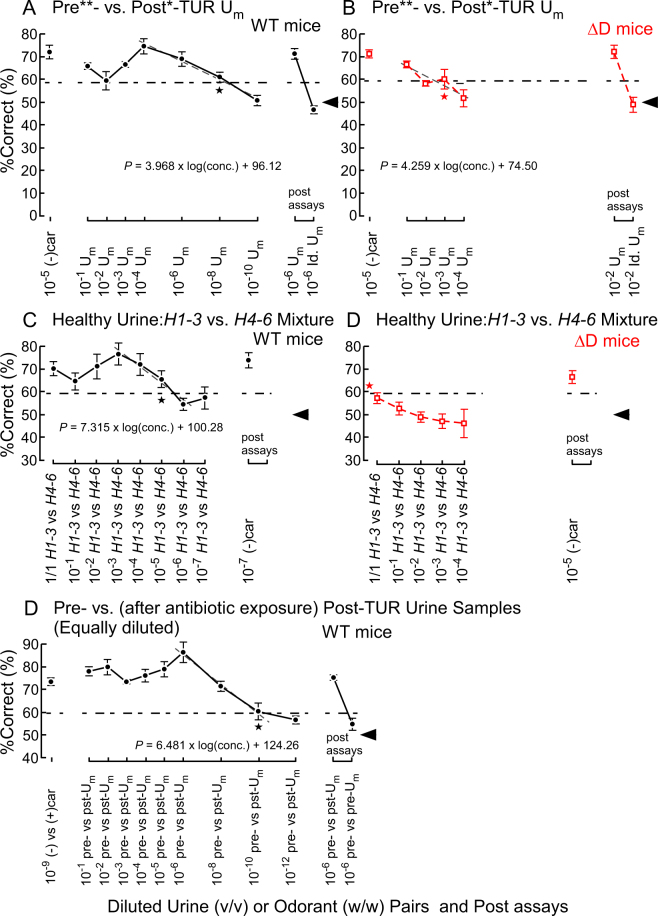
Odour discrimination thresholds of wild-type (WT) and ΔD mice for bladder cancer and healthy volunteer dietary variation. (**A,B**) Odour discrimination of wild-type (WT, black closed circles) and ΔD mice (red open squares) between equi-occult blood pre**- vs. post*-transurethral resection (post*-TUR) urine mixture (U_m_) of five patients with bladder cancer. Post assays, 10^−6^ pre**- vs. post*-TUR U_m_ and identical U_m_ pair: 10^−6^ pre**- vs. pre**-TUR U_m_. The percent correct (%Correct) for a training odour pair—10^−5^ (*R*)-(−)-carvone [(−)car] vs. solvent [di(propylene)glycol] just before the urine discrimination assay is shown on the left side. (**C,D**) Urine odour discrimination between a pair of healthy (*H*) U_m_s—six volunteers’ 1^st^–3^rd^ sample (*H1–3*) vs. 4^th^–6^th^ sample (*H4–6*) U_m_. Post assays: 10^−7^ and 10^−5^ (−)car vs. solvent for WT and ΔD mice, respectively. (**E**) Odour discrimination between equally-diluted pre- vs. (after antibiotic exposure) post-TUR U_m_ of five patients with bladder cancer. Post assay, 10^−6^ pre- vs. post-TUR U_m_ and identical U_m_ pair: 10^−6^ pre- vs. pre-TUR U_m_. The %Correct for a training odour pair: 10^−9^ (−)car vs. (*S*)-(+)-carvone ((+)car) just before the urine discrimination assay is shown on the left side. Two alternative forced choice assays with target vs. non-target odours were performed in a Y-maze. %Correct ± standard error of the mean (SE; 18 trials × 5–7 mice) is shown. Tasks performed at threshold are marked by the star. A linear regression model of %Correct vs. logarithmic concentration (grey broken line) is shown in the range. Chain lines indicate the %Correct significantly above chance performance (*P* = 0.05 for 90 to 126 trials, 18 trials/mouse). Black arrowheads indicate chance levels (50%).

During the initial training in the Y-maze^18^, the average percent correct (%Correct) for the target odour gradually increased higher than chance (example shown in Supplementary Fig. S1, Table ST1). As expected in a 10- or 100-fold dilution series, the %Correct of wild-type (WT) mice declined at lower urine concentrations, resulting in an odour discrimination threshold of 7.7 × 10^−10^ v/v (10^−8^ × 1/13; estimated threshold = 5.7 × 10^−10^ v/v) for a pre- vs. post-transurethral resection (post-TUR) urine mixtures (U_m_s) in equi-occult blood conditions (Table 1; Fig. 1A, Supplementary Tables ST2, ST3). We confirmed the consistency of the choice in the post assays after completing the assays at the lowest concentration at which the sniffer mice could not discriminate the target odours, and were instead rewarded by chance (Fig. 1A).

### Healthy volunteer dietary influence on urine odour is less salient than bladder cancer-induced changes in urine odour

To determine whether dietary variations of healthy volunteer or bladder cancer-induced olfactory cues are more salient in urine samples, we measured the odour discrimination threshold of WT mice for healthy volunteer U_m_ pairs in a 10-fold dilution series in the Y-maze. We obtained an odour discrimination threshold of 1.0 × 10^−5^ v/v (estimated threshold = 2.6 × 10^−6^ v/v) (Table 1; Fig. 1C, Supplementary Tables ST2, ST4). The approximately 10^4^-fold lower discrimination threshold for pre-TUR U_m_ indicates that the urine odour change in patients with bladder cancer is a more salient olfactory cue than healthy volunteer dietary variation. This result also indicates that WT mice are likely insensitive to dietary variation of urine odours at 10^−6^ v/v or lower concentrations. Based on this observation, we propose a dilution of 10^−6^ v/v in equi-occult blood condition to be used in a Y-maze behavioural assay of bladder cancer-induced odour changes in individual patient pre-TUR urine (U_i_) samples.

**Table 1:** Discrimination threshold concentrations of wild-type and ΔD mice for pairs of human urine mixtures in a Y-maze.

Urine mixtures	Dilution types	Discrimination thresholds (D.T.) of urine mixtures		D.T. ratio (ΔD/WT)	Cancer	Stage	Grade (H, high; L, low)	Age	Gender	Sampling period (5 days)
		WT mice	ΔD mice							
pre-TUR *N*:U_m_	equi-occult blood	7.7 × 10^−10^ (5.7 × 10^−10^)	7.7 × 10^−5^ (2.9 × 10^−4^)	5 × 10^5^	bladder	I	3 H + 2 L	69–91	♂	(−53)–(−1)
post-TUR *N*:U_m_					none	—				8–127
pre-TUR *N*:U_m_	equally diluted	1.0 × 10^−10^ (9.9 × 10^−11^)	—	—	bladder	I	3 H + 2 L	69–91	♂	(−53)–(−1)
post-TUR *N*:U_m_					none	—				8–127
pre-TUR *K*:U_m_	equally diluted	1.0 × 10^−13^* (3.2 × 10^−14^)	—	—	bladder	I	4 H + 1 L	69–96	♂	(−60)–(−1)
post-TUR *K*:U_m_					none	—				1–5^†^, 8–123^‡^
healthy *H1–3* U_m_	equally diluted	1.0 × 10^−5^ (2.6 × 10^−6^)	>1.0	>10^5^	none	—	—	52–75	♂	(−17)–(−1)
healthy *H4–6* U_m_					none	—				0–11^#^

WT, wild-type. TUR, transurethral resection. *N*:U_m_, *N*-series urine mixture (U_m_) is an equal-volume mixture of 25 urine samples from five patients: *N6–N10* on five different days. *K*:U_m_, *K*-series U_m_ is an equal-volume mixture of 25 urine samples from five patients: *K3–K5*, *A1*, and *T2* on five different days (four patients^†^; one patient^‡^). Day 0 is the ablative operation day of the patient and the fourth sampling day of the healthy volunteer. Healthy *H1–3* and *H4–6* U_m_s are equal-volume mixtures of 18 urine samples from six healthy volunteers on three different days (*n* = 5, for 1^st^–3^rd^ sampling; *n* = 6, for 4^th^–6^th^ sampling^#^) or exceptionally two different days (*n* = 1, including one-day AM and PM sampling, for 1^st^–3^rd^ sampling). The discrimination thresholds of mice were obtained as the lowest urine concentrations with the average values of the %Correct for 6–7 mice of the same strain (18 trials × 6–7 mice) greater than 59.43% or 58.73% that were significantly different from chance level (*P* = 0.05 for 108 or 126 trials). Estimated threshold concentration in parenthesis was calculated by the odds ratio (%Correct to chance) of the logit, with *P* = 76.65% and linear regression models of %Correct. Urine sample was diluted in distilled water (v/v). *Preliminary result for four mice (the lowest concentration with the %Correct greater than 61.55%, *P* = 0.05 for 72 trials).

### Sniffer WT mice can discriminate between individual pre-TUR U_i_s and post-TUR U_m_ in 10^−6^-fold diluted equi-occult blood conditions

**Figure 2:**
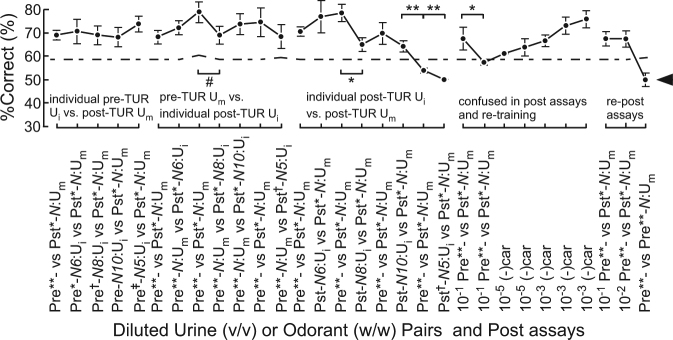
Mice can discriminate urine odours of patients with bladder cancer. Odour discrimination between individual patient pre-transurethral resection (TUR) urine mixture (U_i_) vs. five patient post-TUR urine mixture (U_m_), pre-TUR U_m_ vs. individual patient post-TUR U_i_, and individual patient post-TUR U_i_ vs. post-TUR U_m_. 10^−6^-fold diluted equi-occult blood U_m_s/U_i_s were used except for those indicated otherwise. Two alternative forced choice assays with target vs. non-target odours were performed in a Y-maze. Post assays: 10^−1^ pre- vs. post-TUR U_m_ and identical pair: 10^−6^ pre- vs. pre-TUR U_m_ with re-training. The *P* value of paired difference in %Correct is indicated by ^#^(*P* ≥ 0.05), *(0.01 ≤ *P* < 0.05), and **(*P* < 0.01). %Correct ± standard error of the mean (SE; 18 trials × 5–7 mice) is shown. Extra-dilution rates for equi-occult blood U_m_s were 1/6* v/v, 1/13** v/v, 1/15^†^ v/v, and 1/75^‡^ v/v. Chain lines indicate %Correct significantly above chance performance (*P* = 0.05 for 90 to 126 trials, 18 trials/mouse). Black arrowheads indicate chance levels (50%).

After training with pre-TUR U_m_ (positive control), sniffer WT mice discriminated individual patient pre-TUR U_i_ vs. post-TUR U_m_ in 10^−6^-fold diluted equi-occult blood conditions (left part of Fig. 2, Supplementary Table ST2). The %Correct of WT mice remained almost constant for all four individual patient pre-TUR U_i_ samples and pre-TUR U_m_. This result indicated no significant change in olfactory cues between the individual pre-TUR U_i_ samples and the pre-TUR U_m_ as the bladder cancer-induced urine odour change, even for the *N5* patient U_i_ sample that was not a component of patient U_m_. As described in the next section, it is notable that subsequent assays of two urine samples with different olfactory cues resulted in a significant perturbation in values of %Correct of the sniffer mice.

Next, we tested constancy of the olfactory cue for pre-TUR U_m_ vs. individual post-TUR U_i_, sometimes checking for odour discrimination for the positive control in 10^−6^-fold diluted equi-occult blood conditions (middle left part of Fig. 2, Supplementary Tables ST2, ST3). The %Correct of WT mice remained nearly constant for pre-TUR U_m_ vs. any of the four post-TUR U_i_ samples (no significant difference between two successive performances). This again indicates that dietary and inter-individual variation in individual post-TUR U_i_ samples provided no significant change in olfactory cue of bladder cancer when using conditions that are sub-threshold for dietary variation detection.

Without olfactory cues of bladder cancer, post-TUR urine pairs of individual U_i_ vs. U_m_ confused the sniffer mice, as displayed by significant differences in %Correct between two successive discrimination performances, leading to lowered %Correct even for the positive control U_m_ pair (middle part of Fig. 2, Supplementary Tables ST2, ST3). This indicated that the mice forgot the correct odour choice, so they were re-trained with (–)car and solvent (middle right part of Fig. 2, Supplementary Table ST2). We confirmed the success of re-training by showing a gradually increased %Correct, the ability to discriminate the positive control patient U_m_, and the inability to discriminate identical urine odours in a set of post assays (right part of Fig. 2, Supplementary Table ST2).

### Sniffer WT mice displayed significant perturbation in discrimination performance upon switching olfactory cues between equally diluted and equi-occult blood U_m_s

The odour discrimination threshold of WT mice for equally diluted patient U_m_ pairs that contained occult blood at different concentrations was 1.0 × 10^−10^ v/v (estimated threshold = 9.9 × 10^−11^ v/v; Table 1, Fig. 1E, Supplementary Table ST5). This slightly (5.8-fold estimated) lower discrimination threshold than that for equi-occult blood U_m_ pairs suggests that occult blood markedly alters urinary odours slightly more than bladder cancer. These threshold differences indicate that urinary olfactory cues in U_m_s increase in the following order: dietary variation < bladder cancer < occult blood.

**Figure 3:**
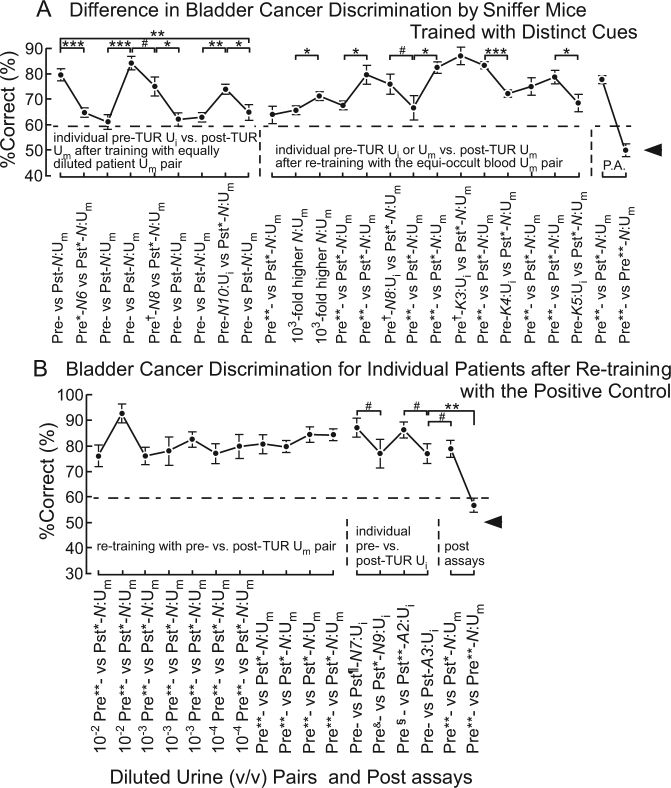
Significant perturbation in odour discrimination performance upon switching olfactory cues between equally diluted and equi-occult blood urine mixtures and reduced perturbation after re-training with the positive control. (**A**) Odour discrimination between equi-occult blood individual patient pre-TUR urine mixture (U_i_) vs. post-TUR U_m_ (on the left part) after training with equally-diluted pre- vs. post-TUR U_m_ and equi-occult blood individual patient pre-TUR U_i_ vs. post-TUR U_m_ (on the middle part) after re-training with equi-occult blood pre- vs. post-TUR U_m_. Post-assay (P.A.): 10^−1^ pre- vs. post-TUR U_m_ and identical U_m_ pair: 10^−6^ pre- vs. pre-TUR U_m_. (**B**) Mice can discriminate individual patient pre- vs. post-TUR urine odours for bladder cancer after re-training with equi-occult blood urine samples. %Correct in odour discrimination between individual patient pre- vs. post-TUR urine mixture (U_i_) are shown. The *P* value of paired difference in %Correct is indicated by ^#^(*P* ≥ 0.05), *(0.01 ≤ *P* < 0.05), **(0.001 ≤ *P* < 0.01) and ***(*P* < 0.001). 10^−6^-fold diluted, equi-occult blood urine samples were used except for those indicated otherwise. Extra-dilution rates were 1/3 v/v, 1/6* v/v, 1/9^§^ v/v, 1/10^&^ v/v, 1/13** v/v, and 1/15^†^ v/v. Chain lines indicate %Correct significantly above chance performance (*P* = 0.05 for 108 trials, 18 trials/mouse). Black arrowheads indicate chance levels (50%).

As expected, sniffer WT mice that were trained with the equally diluted pre- vs. post-TUR U_m_ pair displayed significant %Correct perturbations for odour discrimination of individual pre-TUR U_i_ vs. post-TUR U_m_ in the 10^−6^-fold diluted equi-occult blood conditions (left part of Fig. 3A, Supplementary Tables ST5, ST3), in contrast to the relatively stable %Correct displayed in the left part of Fig. 1. After re-training with the equi-occult blood U_m_ pair, the sniffer WT mice discriminated individual patient pre-TUR U_i_ vs. post-TUR U_m_ (right part of Fig. 3A, Supplementary Fig. S2A, Tables ST5, ST4), similar to WT mice that were initially trained with the equi-occult blood positive control (Fig. 2), except for moderate and smaller perturbations compared to those before re-training. Notably, the sniffer mice were likely not confused after re-training. This change in %Correct stabilities of the same mice confirmed a distinct olfactory cue (occult blood vs. bladder cancer) between equally diluted and equi-occult blood patient U_i_ or U_m_ samples. Furthermore, these results indicate that this Y-maze assay measures the memory-based odour discrimination ability of mice.

Additional experiments confirmed that WT mice discriminated an odour difference between individual pre- and post-TUR U_i_ as the olfactory cues of bladder cancer-induced urine odour disorder (Fig. 3B; Supplementary Tables ST6, ST4). Notably, five patient urine samples (*K3–K5*, *A2*, and *A3*) were not components of the positive control patient U_m_. Together, these results indicate that WT mice discriminated 11/11 individual patient pre-TUR U_i_ samples by the olfactory cue of bladder cancer-induced urine odour change, despite the difference in the occult blood test.

Although only a preliminary result, post-TUR U_m_ collected during antibiotic exposure resulted in an odour discrimination threshold of 1.0 × 10^−13^ v/v (estimated threshold = 3.2 × 10^−14^ v/v), which was the lowest among the four U_m_ pairs tested (Table 1; Supplementary Fig. S2B, Table ST7). The lower threshold suggests that antibiotic drug metabolites alter urine odours more markedly than occult blood. Therefore, observed threshold differences indicate that urinary olfactory cues in U_m_s increase in the following order: dietary variation < bladder cancer < occult blood < antibiotic drug metabolites.

### Sniffer ΔD mice discriminated patient pre-TUR U_m_ with an elevated threshold and did not discriminate between the U_m_s of healthy volunteers

By ablating all dorsal olfactory receptors, the odour discrimination threshold of ΔD mice elevated by 5 × 10^5^-fold in the estimated thresholds (10^−3^ × 1/13 = 7.7 × 10^−5^ v/v; estimated threshold = 2.9 × 10^−4^ v/v) for the equi-occult blood patient pre-TUR U_m_. This reduced discrimination sensitivity of ΔD mice indicates that dorsal olfactory receptors are essential for recognition of body odour disorders (Table 1; Fig. 1B, Supplementary Tables ST2, ST3). Moreover, ΔD mice did not discriminate healthy volunteer U_m_ at the original concentration, indicating a ≥10^5^-fold elevated threshold for dietary variation in body odours and/or the body odours themselves (Table 1; Fig. 1D, Supplementary Table ST2).

## Discussion

**Figure 4:**
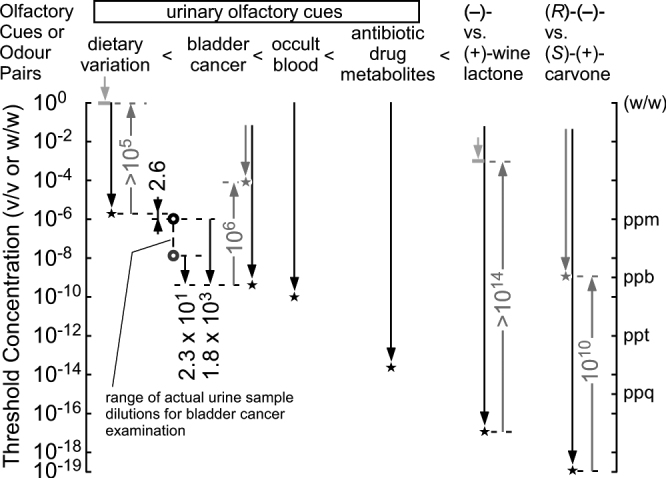
Odour discrimination thresholds of wild-type (WT) and ΔD mice for urinary olfactory cues. Odour discrimination ranges (downward arrows) and thresholds (stars) of WT (black plots) and ΔD mice (red plots) for urinary olfactory cues and enantiomer pairs^18^ are shown. Odour discrimination threshold of ΔD mice for dietary variation of body odours and wine-lactone enantiomers were not observed in the concentration ranges examined (light red arrows), the highest concentrations of which are indicated by the light red bars. Observed threshold differences indicate that urinary olfactory cues increase in the urine mixtures (U_m_s) in the following order: dietary variation < bladder cancer < occult blood < antibiotic drug metabolites. The concentration of 10^−6^ v/v (indicated by the black open circle) is used for bladder cancer examination of individual patient pre-TUR urine samples, as it is sub-threshold for detection of dietary variation in urine and supra-threshold for detecting bladder cancer odours. Actual concentrations of pre-TUR U_m_ samples for bladder cancer examination in 10^−6^-fold diluted equi-occult blood conditions ranged from 10^−6^ v/v (black open circle) to 1.3 × 10^−8^ v/v (grey open circle). ΔD mice exhibited reduced odour discrimination sensitivities compared to WT mice; degrees of sensitivity reduction due to ablation of dorsal olfactory receptors are indicated by the red upward arrows.

In this study, we showed that sniffer mice can discriminate between urine odour changes in patients with bladder cancer compared to dietary and/or inter-individual variations. In the U_m_ condition, in which dietary and inter-individual variations were reduced, urinary olfactory cues of body odour increased in the following order: dietary variation < bladder cancer < occult blood < antibiotic drug metabolites (Fig. 4). This relationship provides a biological basis for detection of body odour disorders in the equi-occult blood U_m_ condition for non-invasive diagnostic tests for cancers or other diseases.

The greater intensity of urinary occult blood olfactory cues compared to genetically determined body odours is consistent with the previous observation that body odour discrimination is more difficult in serum than in urine samples^21^. However, cadaver dogs trained with a series of diluted blood samples can detect different human blood samples even at very low concentrations^22^, suggesting a salient olfactory cue common across individual blood samples. These results emphasise the excellent ability of mice and dogs to discriminate weak but biologically important olfactory cues over those of more abundant compounds.

In humans, the dietary and inter-individual variations among five patients over five days is likely greater than those previously observed in mice with identical genetic background on the same diets, resulting in smaller contribution of each difference to the olfactory cue in U_m_s of 25 samples than two different diets on identical genetically-determined body odours in mice. Indeed, without pre-treatment for dietary and inter-individual variations, sniffer dogs were previously able to detect urine samples from patients with bladder cancer, with a success rate of 41%^15^. Although our sample size was small, the sniffer mice in this study achieved a success rate of 100% for the individual urine samples of 11 patients with or without occult blood, as well as for the positive control U_m_ with occult blood (at a 10^−6^-fold dilution); this was 2.6-fold lower than the discrimination threshold for the urine mixture of healthy volunteers and 23–1800-fold higher than the discrimination threshold for bladder cancer-induced urine odour changes (Fig. 4, estimated thresholds). In a previous study regarding breath samples from patients with lung and breast cancer, sniffer dogs displayed sensitivities of 99% and 88%, respectively^16^. Notably, virus or pathogen infection also specifically alters urine odours in mice on the same diet^23,24^. Therefore, our approach of using sniffer mice to detect urine odour changes in 0.3 nL urine samples (≥0.3 mL × 10^−6^ v/v × extra dilution rate) in equi-occult blood diluted conditions below the detection level of dietary variations may be widely applicable for the diagnosis of other diseases as well as bladder cancer.

Our results first indicate that the proposed Y-maze assay measures the memory-based odour discrimination ability of mice. Furthermore, our results emphasise the need to use proper positive controls when training sniffer mice and dogs. This also requires treatments and experimental designs to maintain fresh urine samples, considering distinct SPME-GC profiles between fresh and aged blood samples^25^. In the present study, the moderate perturbations and lack of confusion in bladder cancer discrimination of re-trained mice observed in this study are likely attributable to weaker target olfactory cues (at a 10^2^-fold higher concentration than the threshold) in re-trained mice (Fig. 3A) compared to that of the initial training (at a 10^7^-fold higher concentration than the threshold) for the first group of mice (Fig. 2). Based on these results, an intense olfactory cue of 10^−1^ v/v or 10^−2^ v/v pre-TUR U_m_ is recommended as the positive control in the initial training of each sniffer mouse.

A limitation of this simple assay is that sniffer mice were able to discriminate a pair of urine samples without the olfactory cue of bladder cancer (middle right part of Fig. 2), possibly reflecting the discrimination of different urine dilution rates. However, repeated assays of the positive control and individual patient pre-TUR U_i_ can be used to identify spurious discrimination of non-target olfactory cues based on no significantly different perturbation in discrimination performance from that observed in switching olfactory cues. An acceptable range of %Correct perturbations will be determined in future studies.

Our results also indicate a possible mechanism underlying the olfactory discrimination of bladder cancer-induced urine odour changes compared to healthy odours. Body odours may evoke similar, yet distinct, odour perceptions through nonlinear contributions of multiple olfactory receptors activated by multiple odorous compounds. Considering that no dietary-specific or tumour-specific odorous compounds appear in SPME-GC-MS profiles^14,19^, it is difficult to explain the discrimination of weaker olfactory cues under the conventional olfactory coding scheme based on simple combinatorial representation of different odours by different subsets of responsive olfactory receptors^26^. The sensory profile of an odour may include several distinct elemental odours if multidimensional input is segmented through parallel pathways^27^.

We previously proposed a mechanism for supersensitive odour discrimination wherein signals from the highly sensitive “helix-8-2^nd^-Glu” dorsal olfactory receptors are integrated with synchronised inputs from cognate olfactory receptors for unique or common elemental odours in third-order neurons^18,28^. This processing is achieved through a feed-forward inhibitory mechanism in the second olfactory centre, the anterior piriform cortex^18,28–33^, via short-latency olfactory bulb (first olfactory centre) tufted cells^34^. A slight change in early feedforward inhibitory signals may alter signal integration of cognate receptors for some elemental odours, thereby changing the elemental odour hierarchy, even if the combination of activated olfactory receptors are identical.

In this study, we found that ΔD mice showed ≥10^5^-fold reduction in discrimination sensitivity for body odours, indicating an essential role of these dorsal receptors in body odour recognition. This reduction is almost half of the 10^10^-fold reduction in carvone enantiomer discrimination sensitivity (Fig. 4)^18^. This difference is likely attributable to a greater number of key olfactory receptors for multiple-compound body odours and/or a major contribution of class I dorsal olfactory receptors, as olfactory receptors for single-compound wine lactone or carvone enantiomers are fewer in number than those of body odours and are mainly comprised of class II olfactory receptors^35^.

In summary, our study provides further evidence for the use of sniffer mice to detect cancer-induced changes in urine odour for the diagnosis of bladder cancer and other diseases. U_m_s successfully enhanced the intensity of the bladder cancer-induced olfactory cues between odour discrimination pairs. Future studies will focus on the accuracy of the examination among various cases and will examine the recurrence risk of bladder cancers, as well as other types of cancers. Currently available analytical instruments are generally less sensitive to the different profiles of trace key compounds in body odours than murine or canine olfactory systems. However, by identifying the trace and abundant key compounds in urine mixtures that occur due to diseases such as bladder cancer, we may be able to determine novel molecular biomarkers for non-invasive disease diagnosis.

## Materials and Methods

### Experimental groups, control groups, and stimuli

This study using human urine samples was approved in accordance with the relative guidelines and Japanese Laws by the Institutional Committee for the Ethics on the Experiments with Human Derivative Samples of Aino University (including urine sampling in Aino University Hospital, Portisland Hospital, Kameoka-Shimizu Hospital, Kyoto-Katsura Hospital and Nozaki Tokushukai Hospital) and the National Institute of Advanced Industrial Science and Technology (Y-maze behavioural assays). All subjects signed informed consent.

In the general population, the number of patients with non-muscle invasive bladder cancer is greater than that of patients with muscle-invasive bladder cancer. In addition, non-muscle invasive bladder cancer is a preferable target for the early diagnosis of bladder cancer. Therefore, we selected patients with non-muscle invasive bladder cancer treated with TUR of the bladder tumour. The subjects included a group of healthy volunteers (*n* = 6, 52–75 years old, six men, *H*-series samples), and two groups of patients with bladder cancer: post-TUR after antibiotics exposure (*n* = 9, 69–91 years old, eight men and one woman, patient IDs: *N5*, *N6*, *N7*, *N8*, *N9*, *N10*, *A1*, *A2*, and *A3*) and post-TUR during antibiotic exposure (*n* = 4, 69–96 years old, four men, patient IDs: *K3*, *K4*, *K5*, and *T2*). After the TUR of bladder tumour, no bladder cancer was detected by cystoscopy in any patients at the three-month mark. In addition, pathological examination also showed that bladder cancer was completely resected. These examinations confirmed that all post-TUR urine samples were collected from patients without bladder cancer.

Urine samples were collected in Aino University Hospital, Portisland Hospital, Kameoka-Shimizu Hospital, Kyoto-Katsura Hospital and Nozaki Tokushukai Hospital from patients or healthy volunteers. Immediately after collection, samples were filtered through 0.2 μm filters and frozen at −20 °C until needed. Diet-influenced urinary olfactory cues were previously shown to be more intense than genetically determined body odours in mice^2^ and patient urine samples were sometimes positive for occult blood (Supplementary Tables ST3, ST4). As the signal averaging of the replicate measures improves the signal-to-noise ratio, mixtures of the replicate urine samples from several patients with bladder cancer would maintain common concentration profiles of compounds to multiple urine samples and reduce relative concentrations of variable compounds among the urine samples. We expected that bladder cancer-induced change in the concentration profile of subsets of compounds would be maintained in mixtures of urine samples from patients with bladder cancer, and that individual-specific or diet-related concentration profile of subsets of compounds would be reduced. This also leads a reduction in possible synergistic or antagonistic effects of relatively-diluted variable compounds on bladder cancer-related odours in the urine sample mixtures.

We used urine mixtures (U_m_s) of 25 equal-volume urine samples (5 patients × 5 samples) that were collected from five patients on five different days, respectively, in each pre- and post-TUR condition. To reduce the influence of occult blood on urinary olfactory cues, we prepared patient pre- vs. post-TUR U_m_s at equal concentrations of occult blood (defined here as equi-occult blood) in a 10- or 100-fold dilution series. Extra-dilution rates for the equi-occult blood condition (adjusting for 0.01 mg/dL or less haemoglobin in the stocked U_m_s or U_i_s samples) were determined on the basis of haemoglobin concentrations roughly estimated using test strips (Uri-Ace Kc, Terumo Corp., Tokyo, Japan; Supplementary Tables ST3, ST4).

For an olfactory cue of bladder cancer, equi-occult blood, pre- and post-TUR U_m_ after antibiotics exposure were prepared with urine samples from five patients (69–91 years old, five men [*N6*, *N7*, *N8*, *N9*, and *N10*], stage = I, grade = high for three patients and low for two patients) on five different days. The 10^−6^-diluted, equi-occult blood pre- and post-TUR U_m_ pair was used as the positive control. For an olfactory cue of occult blood, we measured the odour discrimination threshold of sniffer mice for equally-diluted pre- vs. post-TUR U_m_ pair after antibiotics exposure for comparison with that of the equi-occult blood pre- vs. post-TUR U_m_ pair in a Y-maze behavioural assay (see the behavioural assay section). If an equally-diluted pre- vs. post-TUR U_m_ pair presents occult blood-related odour as the most salient olfactory cue, then (i) the odour discrimination threshold of mice for the equally-diluted pre- vs. post-TUR U_m_ pair must be lower than that of the equi-occult blood U_m_ pair, and (ii) odour presentation alternatively switching salient olfactory cues between equally-diluted and equi-occult blood U_m_ pairs would confuse the decisions of behaving mice. Individual patient urine mixtures (U_i_s) of five equal-volume urine samples were used to examine differences in urinary olfactory cues between pre- and post-TUR urine samples in individual patients.

For an olfactory cue of diet-influenced urine samples in healthy volunteers, we prepared two U_m_s that each contained 18 equal-volume urine samples (6 volunteers × 3 samples) that were collected from six volunteers on six different days (*H1–3*: 1st–3rd sampling days, and *H4–6*: 4th–6th sampling days), and one volunteer on five different days including 1-day AM and PM sampling. For an olfactory cue of antibiotic drug metabolites, post-TUR U_m_ during antibiotics exposure was prepared with urine samples from five patients including one patient after antibiotic exposure (69–96 years old, five men [*K3*, *K4*, *K5*, *T2*, and *A1*], stage = I, grade = high for four patients and low for one patient) on five different days.

### Wild-type and ΔD mice

All experimental procedures were approved by the Institutional Animal Care and Use Committees of Osaka Bioscience Institute, Kansai Medical University, and the National Institute of Advanced Industrial Science and Technology, and are in accordance with the Japanese Law for the Humane Treatment and Management of Animals (No. 105) and Japanese Fundamental Guidelines for Proper Conduct of Animal Experiment and Related Activities in Academic Research Institutions under the jurisdiction of the Ministry of Education, Culture, Sports, Science and Technology. ΔD mice (mixed background of C57BL/6 and 129Svj) were obtained by crossing *O-MACS-Cre* mice, in which the *Cre* recombinase gene was inserted in-frame into the ATG site of the *O-MACS* gene^20^, and *Eno2-STOP-DTA* mice, in which the Cre-inducible *diphtheria toxin A* gene was introduced into the *neuron-specific enolase* gene^36^. Seven ΔD male mice and 23 WT male mice (C57BL/6 CrN Slc, SLC Japan, Inc., Hamamatsu, Japan) were narrowed to six sniffer ΔD and 17 sniffer WT mice, respectively, that actively worked with a trainer.

### Y-maze behavioural assay

We conducted two alternative forced choice behavioural assays in a Y-maze to measure odour discrimination thresholds of sniffer mice in a 10- or 100-fold dilution series. A negative pressure-guided odour plume-like flow in the Y-maze enabled us to measure discrimination thresholds lower than ppq levels for single compound enantiomers^18^.

The initial training started with 3-week old mice in the order of habituation to a trainer, Y-maze, and drinking from a small glass funnel for water reward in the Y-maze. Each mouse was then trained to choose a target odour of 10^−3^–10^−9^-w/w (R)-(−)-carvone vs. solvent [di(propylene)glycol] or (S)-(+)-carvone until the average %Correct for the target odour increased to approximately 80%. When mice chose to run in the arm of non-target odour, the terminal caps with the small glass funnel for water reward was immediately removed to prevent mice from drinking the water. The terminal caps with cotton balls absorbing 0.3-mL odour solution and the small glass funnel were independently and randomly exchanged between the two arms. This ensured that mice evenly selected one of the two arms when identical odours were presented in both arms. Each sniffer mouse then explored each urine odour at the same dilution rate with or without respective extra-dilutions in a set of 18 successive trials each day for one or two days. The %Correct on the second day in the two-day assays was analysed for odour discrimination thresholds, because the %Correct on the first day was sometimes perturbed by changes in odour intensities compared to those of the previous days. The animals were deprived of water for 1 day prior to the behavioural assays and were then provided 1–3 mL water daily or given free access to water for 30–60 s after the assays. Further details have been described previously^18^.

The discrimination threshold was defined as the lowest concentration of diluted urine samples at which the average %Correct for the target odour was significantly higher than chance. To confirm the consistency of the odour choice, sniffer mice were tested after completing the assays at the lowest concentration to determine if they were able to: (i) select the target odour (pre-TUR U_m_/U_i_ or an odorant) vs. non-target odour (post-TUR U_m_/U_i_ or the solvent), at one of the discriminative concentrations and (ii) select one of two identical odours (IO) by chance.

### Statistical Analysis

The %Correct for one of two identical targets is 50% (chance) in the two-alternative choice task. Statistical analyses of average %Correct among all mice for individual odour pairs at respective concentrations were performed using the chi-square test for total numbers of trials (e.g., > 59.43%, *P* < 0.05 for 108 trials). The Smirnov-Grubbs’ test was used to detect outliers. Only an outlier of 38.9^#^ (statistic T = 2.384 > T_0_ = 2.285, *P* = 0.05, one-way) among 12 data^&^ for U_i_ of *K5* was detected and excluded from analysis. Notably, a mouse (*wt8*) exhibited an inactive behaviour in this case. *P* values (Student’s t-test) in statistical comparisons of two successive or selected %Correct of WT mice was calculated using Microsoft Excel 2013. Estimated threshold concentrations were calculated as the concentrations for the odds ratio (%Correct to chance), 59.43% ÷ 50% = 1.1886 of the logit, with *P* = 76.65% by linear regression models of %Correct vs. logarithmic concentration for the ranges as shown in Fig. 1.

## Electronic supplementary material

Supplementary Information
