# Supplementary material for: Sniffer mice discriminate urine odours of patients with bladder cancer: A proof-of-principle study for non-invasive diagnosis of cancer-induced odours
Source: Sci Rep. 2017 Nov 7;7:14628. doi: 10.1038/s41598-017-15355-z (PMC5676727; doi:10.1038/s41598-017-15355-z)
Supplement: Supplementary file 1 — Supplementary Information [file 41598_2017_15355_MOESM1_ESM.pdf]

Supplementary Information

# Sniffer mice discriminate urine odours of patients with bladder cancer: A proof-of-principle study for non-invasive diagnosis of cancer-induced odours

***Takaaki Sato[1], Yoji Katsuoka[2], Kimihiko Yoneda[3], Mitsuo Nonomura[4], Shinya Uchimoto[5], Reiko Kobayakawa[6], Ko Kobayakawa[6], Yoichi Mizutani[7]***

*[1]Biomedical Research Institute, National Institute of Advanced Industrial Science and Technology, Osaka 563-8577, Japan; [2] Department of Urology, Portisland Hospital, Hyogo 650-0046, Japan; [3] Department of Urology, Kameoka-Shimizu Hospital, Kyoto 621-0834, Japan; [4] Department of Urology, Kyoto-Katsura Hospital, Kyoto 615-8256, Japan; [5] Department of Urology, Nozaki Tokushukai Hospital, Osaka 574-0074, Japan; [6] Institute of Biomedical Science, Kansai Medical University, Hirakata, Osaka 573-1010, Japan; [7] Department of Medical Engineering, Faculty of Health Science, Aino University, Osaka 567-0012, Japan*

## Tables and Figure Legends

**Fig. S1.** Initial training of wild-type (WT) and ΔD mice for odour discrimination. (*A*), %Correct of WT mice (black closed circles). (*B*), %Correct of ΔD mice (red open squares) that genetically lost all dorsal olfactory receptors. Two alternative forced choice assays with target vs. non-target odorus were performed in a Y-maze. %Correct ± standard error of the mean (SE; 6–24 trials × 4–7 mice) is shown. Broken lines indicate the %Correct significantly above chance performance ($P$ = 0.05 for 24 to 144 trials, 6–24 trials/mouse). Black arrowheads indicate chance levels (50%).

**Table ST1.** %Correct of wild-type (WT) and ΔD mice in initial training for odour discrimination in a Y-maze.
Each WT mouse was performed twelve to twenty four trials of odour discrimination per day with an award of a drop of water for the concordance odour choice. The %Correct for $P$ = 0.05 level was calculated for 36–126 trials (indicated by the chain line in Supplementary Fig. S1). SE, standard error.
**Table ST1.** %Correct of WT and ΔD mice in initial training for odour discrimination in a Y-maze (continued).

Each ΔD mouse was performed six to twenty four trials of odour discrimination per day with an award of a drop of water for the concordance odour choice. The %Correct for $P$ = 0.05 level were calculated for 24–144 trials (indicated by the chain line in Supplementary Fig. S1). SE, standard error.

**Table ST2.** %Correct of wild-type (WT) and ΔD mice for serial 10- or 100-fold diluted equi-occult blood urine samples in a Y-maze.
Extra-dilution rates for equi-occult blood urine samples were 1/6* v/v, 1/13** v/v, 1/15† v/v, and 1/75‡ v/v. SE, standard error.
**Table ST2.** %Correct of WT and ΔD mice for serial 10- or 100-fold diluted equi-occult blood urine samples in a Y-maze (continued).
Extra-dilution rates for equi-occult blood urine samples were 1/6* v/v and 1/13** v/v. (–)car, (*R*)-(–)-carvone (w/w); solvent, di(propylene)glycol. SE, standard error.

**Table ST3.** Blood, proteins, glucose testing in N-series patient urine samples using urine test strips.
$U_i$, individual patient urine mixture ($U_i$) of equal volumes of five urine samples from each patient. $N$:$U_m$, *N*-series urine mixture ($U_m$) of equal volumes of 25 urine samples from five patient *N6–N10* on five different days for each pre- and post-transurethral resection (post-TUR) after antibiotic exposure. Abbreviation: blad., bladder. Ranges of patient 1st–5th sampling days are shown, when day 0 is the ablative operation day of the patient.

**Table ST4.** Blood, proteins, glucose testing in *K*- and *A*-series patient and healthy volunteer urine samples using urine test strips.
$U_i$, individual patient urine mixture ($U_i$) of equal volumes of five urine samples from each patient. $K$:$U_m$, *K*-series urine mixture ($U_m$) of equal volumes of 25 urine samples from five patient *K3–K5, A1* and *T2* on five different days for each pre-transurethral resection (pre-TUR) and antibiotic-exposure post-TUR except for a patient (four patients* + one patient**); Extra-dilution rates were determined by using linear regression of Hb = (0.15, 0.03)† for diluted urine = ($10^{-1}$, $10^{-2}$). *H1–3,* an equal-volume mixture of 18 urine samples from six healthy volunteer on three (n = 5) or two (n = 1, including one-day AM and PM sampling) different days; *H4–6*, an equal-volume mixture of 18 urine samples from the identical six healthy volunteers on three (n = 6) different days. Ranges of patient 1st–5th, healthy volunteer 1st–3rd‡ and 4th–6th¶ sampling days are shown, when day 0 is the ablative operation day of the patient or the fourth sampling day of the healthy volunteer.

**Fig. S2.** Urine odour discrimination performance of wild-type (WT) mice for individual patient urine mixtures ($U_i$) and antibiotic drug metabolite-containing urine mixtures ($U_m$) in the Y-maze. (*A*), Odour discrimination between equi-occult blood individual patient pre-transurethral resection (TUR) $U_i$ vs. post-TUR $U_m$ after re-training with the equi-occult blood pre- vs. post-TUR $U_m$. Extra-dilution rates for equi-occult blood $U_m$s were 1/6* v/v, 1/9§ v/v, 1/10& v/v, and 1/13** v/v. *P* value of paired difference in %Correct is indicated by #($P \geq 0.05$, not significant) and **($0.001 \leq P < 0.01$). (*B*), Odour discrimination between equally-diluted pre- vs. (during antibiotic exposure) post-TUR $U_m$ of other five patients with bladder cancer. Post-assay, $10^{-3}$ pre- vs. post-TUR $U_m$ and identical $U_m$ pair: $10^{-3}$ pre- vs. pre-TUR $U_m$. Tasks performed at thresholds are marked by the star. Chain lines indicate the %Correct significantly above chance performance ($P$ = 0.05 for 90 to 126 trials, 18 trials/mouse). Black arrowheads indicate chance levels (50%).

**Table ST5.** %Correct of wild-type (WT) for serial diluted urine samples in a Y-maze. Extra-dilution rates for equi-occult blood urine samples were 1/6* v/v, 1/9§ v/v, 1/10& v/v, 1/13** v/v, and 1/15† v/v. (+)-car, (S)-(+)-carvone (w/w). An outlier of %Correct = 38.9# (statistic T = 2.384 > $T_0$ = 2.285, $P$ = 0.05, one-way) among 12 data※ for *K5*:$U_i$ was excluded from analysis. *P* values of paired difference in %Correct was calculated by using the Student’s t-test. ‡*P* value is for the *N*:$U_m$ pair of the first and last %Correct, and ¶*P* value is for the pair of the *K5*:$U_i$ and last identical $U_m$ %Correct. SE, standard error.

**Table ST6.** %Correct of wild-type (WT) mice for re-training and individual patient pre- and post-transurethral resection (post-TUR) urine mixture ($U_i$) discrimination.
Extra-dilution rates for equi-occult blood urine samples were 1/3¶ v/v, 1/6* v/v, 1/9§ v/v, 1/10& v/v, and 1/13** v/v. *P* value is for the pair of successive %Correct. SE, standard error.

**Table ST7.** %Correct of wild-type (WT) mice for serial 10-fold diluted mixtures of urine samples collected during antibiotic exposure after transurethral resection (post-TUR).
SE, standard error.

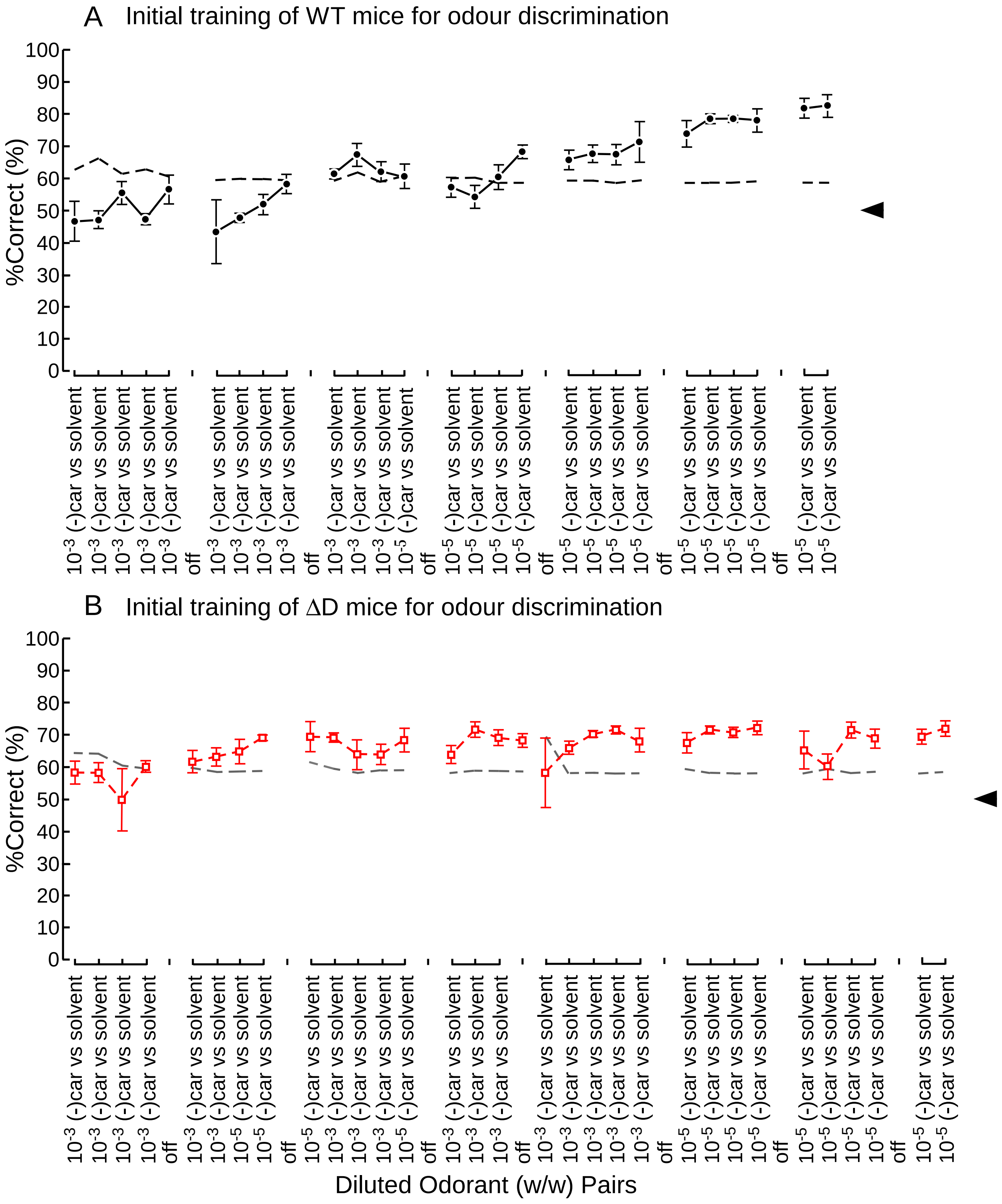


**Fig. S1.** Initial training of wild-type (WT) and ΔD mice for odour discrimination. (*A*), The %Correct of WT mice (black closed circles). (*B*), The %Correct of ΔD mice (red open squares) that genetically lost all dorsal olfactory receptors. Two alternative forced choice assays with target vs. non-target odours were performed in a Y-maze. %Correct ± standard error of the mean (SE; 6-24 trials x 4-7 mice) is shown. Broken lines indicate the %Correct significantly above chance performance ($P$ = 0.05 for 24 to 144 trials, 6-24 trials/mouse). Black arrowheads indicate chance levels (50%).

**Table ST1.** %Correct of wild-type (WT) and ΔD mice in initial training for odour discrimination in a Y-maze.

| Odour pairs | %Correct of individual WT mice (%) | | | | | | total %Correct | avg. of 6 WT mice | SE | %Correct for *P* = 0.05 |
|---|---|---|---|---|---|---|---|---|---|---|
| | *wt8* | *wt9* | *wt10* | *wt11* | *wt12* | *wt13* | | | | |
| Initial training for odour discrimination | | | | | | | | | | |
| $10^{-3}$ (-)car vs. solvent | 33.3 | | 33.3 | 50.0 | 50.0 | 66.7 | 28/60 | 46.7 | 6.2 | 62.7 |
| $10^{-3}$ (-)car vs. solvent | 41.7 | 50.0 | | 50.0 | | | 17/36 | 47.2 | 2.8 | 66.3 |
| $10^{-3}$ (-)car vs. solvent | 58.3 | 41.7 | 58.3 | 58.3 | 50.0 | 66.7 | 40/72 | 55.6 | 3.5 | 61.5 |
| $10^{-3}$ (-)car vs. solvent | 50.0 | 50.0 | 50.0 | 41.7 | 45.5 | | 28/59 | 47.5 | 1.7 | 62.8 |
| $10^{-3}$ (-)car vs. solvent | 50.0 | 61.1 | 44.4 | 66.7 | | 61.1 | 47/84 | 56.0 | 4.5 | 60.7 |
| off | | | | | | | | | | |
| $10^{-3}$ (-)car vs. solvent | 38.9 | 44.4 | 72.2 | 55.6 | 50.0 | | 47/108 | 43.5 | 9.9 | 59.4 |
| $10^{-3}$ (-)car vs. solvent | 50.0 | 50.0 | 55.0 | | 55.6 | 50.0 | 47/98 | 48.0 | 1.4 | 59.9 |
| $10^{-3}$ (-)car vs. solvent | 58.3 | 54.2 | 41.7 | | 50.0 | 50.0 | 52/100 | 52.0 | 3.2 | 59.8 |
| $10^{-3}$ (-)car vs. solvent | 50.0 | 54.2 | 58.3 | 66.7 | 66.7 | 66.7 | 63/108 | 58.3 | 3.0 | 59.4 |
| off | | | | | | | | | | |
| $10^{-3}$ (-)car vs. solvent | 58.3 | 58.3 | 58.3 | 66.7 | 66.7 | 60.0 | 70/116 | 60.3 | 1.7 | 59.1 |
| $10^{-3}$ (-)car vs. solvent | 66.7 | 66.7 | 83.3 | 55.0 | 65.0 | 66.7 | 45/70 | 64.3 | 3.7 | 61.7 |
| $10^{-3}$ (-)car vs. solvent | 58.3 | 54.2 | 58.3 | 75.0 | 60.0 | 66.7 | 74/120 | 61.7 | 3.1 | 58.9 |
| $10^{-5}$ (-)car vs. solvent | 62.5 | 54.2 | 70.8 | 55.0 | | | 56/92 | 57.3 | 3.9 | 60.2 |
| off | | | | | | | | | | |
| $10^{-5}$ (-)car vs. solvent | 54.2 | 54.2 | 62.5 | 66.7 | 50.0 | | 55/96 | 54.3 | 3.1 | 60.0 |
| $10^{-5}$ (-)car vs. solvent | 62.5 | 54.2 | 54.2 | 45.0 | | | 50/92 | 60.5 | 3.6 | 60.2 |
| $10^{-5}$ (-)car vs. solvent | 66.7 | 66.7 | 62.5 | 41.7 | 60.0 | 55.0 | 75/124 | 68.3 | 3.9 | 58.8 |
| $10^{-5}$ (-)car vs. solvent | 70.8 | 70.8 | 62.5 | 61.1 | 72.2 | 72.2 | 86/126 | 65.7 | 2.1 | 58.7 |
| off | | | | | | | | | | |
| $10^{-5}$ (-)car vs. solvent | 62.5 | 58.3 | 66.7 | 77.8 | 66.7 | | 71/108 | 66.4 | 3.0 | 59.4 |
| $10^{-5}$ (-)car vs. solvent | 61.1 | 66.7 | 61.1 | 77.8 | 66.7 | 72.2 | 73/108 | 67.5 | 2.7 | 59.4 |
| $10^{-5}$ (-)car vs. solvent | 62.5 | 58.3 | 66.7 | 77.8 | 66.7 | 77.8 | 85/126 | 68.3 | 3.3 | 58.7 |
| $10^{-5}$ (-)car vs. solvent | 70.8 | 66.7 | 75.0 | 100.0 | 58.3 | 58.3 | 77/108 | 71.3 | 6.3 | 59.4 |
| off | | | | | | | | | | |
| $10^{-5}$ (-)car vs. solvent | 70.8 | 70.8 | 66.7 | 94.4 | 72.2 | 72.2 | 93/126 | 73.8 | 4.1 | 58.7 |
| $10^{-5}$ (-)car vs. solvent | 83.3 | 75.0 | 75.0 | 83.3 | 77.8 | 77.8 | 99/126 | 78.6 | 1.5 | 58.7 |
| $10^{-5}$ (-)car vs. solvent | 83.3 | 79.2 | 75.0 | 77.8 | 77.8 | 77.8 | 99/126 | 78.6 | 1.1 | 58.7 |
| $10^{-5}$ (-)car vs. solvent | 83.3 | 75.0 | 79.2 | 72.2 | 66.7 | 91.7 | 89/114 | 78.1 | 3.6 | 59.2 |
| off | | | | | | | | | | |
| $10^{-5}$ (-)car vs. solvent | 91.7 | 79.2 | 70.8 | 88.9 | 83.3 | 77.8 | 103/126 | 81.7 | 3.1 | 58.7 |
| $10^{-5}$ (-)car vs. solvent | 87.5 | 70.8 | 79.2 | 88.9 | 94.4 | 77.8 | 104/126 | 82.5 | 3.5 | 58.7 |

Each WT mouse was performed twelve to twenty four trials of odour discrimination per day with an award of a drop of water for the concordance odour choice. The %Correct for *P* = 0.05 level were calculated for 36–126 trials (indicated by the chain line in Supplementary Fig. S1). SE, standard error.

**Table ST1.** %Correct of WT and ΔD mice in initial training for odour discrimination in a Y-maze (continued).

| Odour pairs | %Correct of individual ΔD mice (%) | | | | | | total %Correct | avg. of 6 ΔD mice | SE | %Correct for *P* = 0.05 |
|---|---|---|---|---|---|---|---|---|---|---|
| | *ΔD1* | *ΔD2* | *ΔD3* | *ΔD4* | *ΔD5* | *ΔD6* | | | | |
| Initial training for odour discrimination | | | | | | | | | | |
| $10^{-3}$ (-)car vs. solvent | 50.0 | 50.0 | 66.7 | 66.7 | 66.7 | 66.7 | 28/48 | 58.3 | 3.5 | 64.1 |
| $10^{-3}$ (-)car vs. solvent | 58.3 | 58.3 | 66.7 | 66.7 | 50.0 | 50.0 | 28/48 | 58.3 | 3.0 | 64.1 |
| $10^{-3}$ (-)car vs. solvent | 70.0 | 0.0 | 50.0 | 44.4 | 50.0 | 55.6 | 43/86 | 50.0 | 9.7 | 60.6 |
| $10^{-3}$ (-)car vs. solvent | 66.7 | 61.1 | 55.6 | 61.1 | 55.6 | 61.1 | 65/108 | 60.2 | 1.7 | 59.4 |
| off | | | | | | | | | | |
| $10^{-3}$ (-)car vs. solvent | 77.8 | 55.6 | 60.0 | 55.0 | 60.0 | 60.0 | 66/107 | 61.7 | 3.4 | 59.5 |
| $10^{-3}$ (-)car vs. solvent | 62.5 | 50.0 | 62.5 | 66.7 | 62.5 | 70.8 | 81/128 | 63.3 | 2.8 | 58.7 |
| $10^{-5}$ (-)car vs. solvent | 56.3 | 50.0 | 66.7 | 70.8 | 62.5 | 75.0 | 83/128 | 64.8 | 3.8 | 58.7 |
| $10^{-5}$ (-)car vs. solvent | 66.7 | 66.7 | 70.8 | 70.8 | 66.7 | 70.8 | 83/120 | 69.2 | 0.9 | 58.9 |
| off | | | | | | | | | | |
| $10^{-5}$ (-)car vs. solvent | 66.7 | 58.3 | 83.3 | 83.3 | 58.3 | 66.7 | 50/72 | 69.4 | 4.6 | 61.5 |
| $10^{-5}$ (-)car vs. solvent | 66.7 | 66.7 | 70.0 | 75.0 | 70.0 | 65.0 | 72/104 | 69.2 | 1.5 | 59.6 |
| $10^{-3}$ (-)car vs. solvent | 66.7 | 66.7 | 75.0 | 66.7 | 41.7 | 66.7 | 92/144 | 63.9 | 4.6 | 58.2 |
| $10^{-3}$ (-)car vs. solvent | 70.8 | 66.7 | 65.0 | 60.0 | 50.0 | 70.0 | 78/122 | 63.9 | 3.2 | 58.9 |
| $10^{-3}$ (-)car vs. solvent | 70.8 | 66.7 | 77.8 | 77.8 | 55.6 | 61.1 | 78/114 | 68.4 | 3.7 | 59.2 |
| off | | | | | | | | | | |
| $10^{-3}$ (-)car vs. solvent | 70.8 | 66.7 | 70.8 | 62.5 | 54.2 | 58.3 | 92/144 | 63.9 | 2.8 | 58.2 |
| $10^{-3}$ (-)car vs. solvent | 75.0 | 66.7 | 70.8 | 66.7 | 79.2 | | 86/120 | 71.7 | 2.4 | 58.9 |
| $10^{-3}$ (-)car vs. solvent | 75.0 | 66.7 | 66.7 | 62.5 | 75.0 | | 83/120 | 69.2 | 2.5 | 58.9 |
| off | | | | | | | | | | |
| $10^{-3}$ (-)car vs. solvent | 83.3 | 66.7 | 33.3 | 0.0 | 50.0 | | 14/24 | 58.3 | 10.8 | 70.0 |
| $10^{-3}$ (-)car vs. solvent | 70.8 | 66.7 | 66.7 | 70.8 | 62.5 | 58.3 | 95/144 | 66.0 | 2.0 | 58.2 |
| $10^{-3}$ (-)car vs. solvent | 70.8 | 70.8 | 70.8 | 70.8 | 70.8 | 66.7 | 101/144 | 70.1 | 0.7 | 58.2 |
| $10^{-3}$ (-)car vs. solvent | 70.8 | 66.7 | 75.0 | 75.0 | 70.8 | 70.8 | 103/144 | 71.5 | 1.3 | 58.2 |
| $10^{-3}$ (-)car vs. solvent | 79.2 | 70.8 | 62.5 | 58.3 | 75.0 | 62.5 | 98/144 | 68.1 | 3.3 | 58.2 |
| off | | | | | | | | | | |
| $10^{-5}$ (-)car vs. solvent | 75.0 | 75.0 | 75.0 | 62.5 | 58.3 | 62.5 | 73/108 | 67.6 | 3.2 | 59.4 |
| $10^{-5}$ (-)car vs. solvent | 75.0 | 70.8 | 75.0 | 66.7 | 70.8 | 70.8 | 103/144 | 71.5 | 1.3 | 58.2 |
| $10^{-5}$ (-)car vs. solvent | 75.0 | 70.8 | 75.0 | 66.7 | 70.8 | 66.7 | 102/144 | 70.8 | 1.5 | 58.2 |
| $10^{-5}$ (-)car vs. solvent | 79.2 | 70.8 | 75.0 | 66.7 | 75.0 | 66.7 | 104/144 | 72.2 | 2.1 | 58.2 |
| off | | | | | | | | | | |
| $10^{-5}$ (-)car vs. solvent | 79.2 | 79.2 | 58.3 | 45.8 | 75.0 | 54.2 | 94/144 | 65.3 | 5.9 | 58.2 |
| $10^{-5}$ (-)car vs. solvent | 75.0 | 58.3 | 70.8 | 54.2 | 58.3 | 50.0 | 65/108 | 60.2 | 4.0 | 59.4 |
| $10^{-5}$ (-)car vs. solvent | 79.2 | 75.0 | 75.0 | 62.5 | 70.8 | 66.7 | 103/144 | 71.5 | 2.5 | 58.2 |
| $10^{-5}$ (-)car vs. solvent | 79.2 | 70.8 | 66.7 | 62.5 | 70.8 | 58.3 | 91/132 | 68.9 | 3.0 | 58.5 |
| off | | | | | | | | | | |
| $10^{-5}$ (-)car vs. solvent | 79.2 | 70.8 | 66.7 | 66.7 | 70.8 | 62.5 | 100/144 | 69.4 | 2.3 | 58.2 |
| $10^{-5}$ (-)car vs. solvent | 61.1 | 77.8 | 70.8 | 70.8 | 75.0 | 75.0 | 95/132 | 72.0 | 2.4 | 58.5 |

Each ΔD mouse was performed six to twenty four trials of odour discrimination per day with an award of a drop of water for the concordance odour choice. The %Correct for *P* = 0.05 level were calculated for 24–144 trials (indicated by the chain line in Supplementary Fig. S1). SE, standard error.

**Table ST2.** %Correct of wild-type (WT) and ΔD mice for serial 10- or 100-fold diluted equi-occult blood urine samples in a Y-maze.

| Odour pairs | %Correct of individual WT mice (%) | | | | | | | avg. of 5-7 WT mice | SE | %Correct for $P$ = 0.05 |
|---|---|---|---|---|---|---|---|---|---|---|
| | *wt1* | *wt2* | *wt3* | *wt4* | *wt5* | *wt6* | *wt7* | | | |
| Pre**- vs. post*-TUR urine mixture ($U_m$), equi-occult blood | | | | | | | | | | |
| $10^{-5}$ (-)car vs. solvent | 66.7 | 83.3 | 66.7 | 83.3 | 72.2 | 66.7 | 66.7 | 72.2 | 3.0 | 58.7 |
| $10^{-1}$ pre**- vs. post*-N :$U_m$ | 61.1 | 66.7 | 66.7 | 72.2 | 66.7 | 61.1 | 66.7 | 65.9 | 1.4 | 58.7 |
| $10^{-2}$ pre**- vs. post*-N :$U_m$ | 61.1 | 66.7 | 44.4 | 66.7 | 66.7 | 44.4 | 66.7 | 59.5 | 4.0 | 58.7 |
| $10^{-3}$ pre**- vs. post*-N :$U_m$ | 66.7 | 66.7 | 66.7 | 66.7 | 66.7 | 72.2 | 61.1 | 66.7 | 1.2 | 58.7 |
| $10^{-4}$ pre**- vs. post*-N :$U_m$ | 72.2 | 72.2 | 72.2 | 94.4 | 72.2 | 66.7 | 72.2 | 74.6 | 3.4 | 58.7 |
| $10^{-6}$ pre**- vs. post*-N :$U_m$ | 66.7 | 77.8 | 61.1 | 77.8 | 72.2 | 55.6 | 72.2 | 69.0 | 3.2 | 58.7 |
| $10^{-8}$ pre**- vs. post*-N :$U_m$ | 55.6 | 61.1 | 55.6 | 61.1 | 72.2 | 61.1 | 61.1 | 61.1 | 2.1 | 58.7 |
| $10^{-10}$ pre**- vs. post*-N :$U_m$ | 44.4 | 50.0 | 55.6 | 50.0 | 44.4 | 50.0 | 61.1 | 50.8 | 2.2 | 58.7 |
| Post assays | | | | | | | | | | |
| $10^{-6}$ pre**- vs. post*-N :$U_m$ | 66.7 | 83.3 | 66.7 | 72.2 | 72.2 | 72.2 | 66.7 | 71.4 | 2.2 | 58.7 |
| $10^{-6}$ pre**- vs. pre**-N :$U_m$ | 50.0 | 50.0 | 50.0 | 44.4 | 44.4 | 50.0 | 38.9 | 46.8 | 1.7 | 58.7 |
| Healthy volunteer $1^{st}$–$3^{rd}$ $U_m$ (*H1–3*) vs. $4^{th}$–$6^{th}$ $U_m$ (*H4–6*) | | | | | | | | | | |
| *H1–3* vs. *H4–6* $U_m$ | 72.2 | 72.2 | 83.3 | 66.7 | 61.1 | 66.7 | | 70.4 | 3.1 | 59.4 |
| $10^{-1}$ *H1–3* vs. *H4–6* $U_m$ | 55.6 | 61.1 | 77.8 | 66.7 | 55.6 | 72.2 | | 64.8 | 3.7 | 59.4 |
| $10^{-2}$ *H1–3* vs. *H4–6* $U_m$ | 77.8 | 88.9 | 55.6 | 77.8 | 72.2 | 55.6 | | 71.3 | 5.4 | 59.4 |
| $10^{-3}$ *H1–3* vs. *H4–6* $U_m$ | 77.8 | 83.3 | 66.7 | 94.4 | 77.8 | 61.1 | | 76.9 | 4.8 | 59.4 |
| $10^{-4}$ *H1–3* vs. *H4–6* $U_m$ | 83.3 | 83.3 | 55.6 | 72.2 | 77.8 | 61.1 | | 72.2 | 4.8 | 59.4 |
| $10^{-5}$ *H1–3* vs. *H4–6* $U_m$ | 72.2 | 72.2 | 50.0 | 72.2 | 66.7 | 61.1 | | 65.7 | 3.6 | 59.4 |
| $10^{-6}$ *H1–3* vs. *H4–6* $U_m$ | 61.1 | 61.1 | 44.4 | 55.6 | 55.6 | 50.0 | | 54.6 | 2.7 | 59.4 |
| $10^{-7}$ *H1–3* vs. *H4–6* $U_m$ | 50.0 | 61.1 | 44.4 | 61.1 | 77.8 | 50.0 | | 57.4 | 4.9 | 59.4 |
| Post assays | | | | | | | | | | |
| $10^{-7}$ (-)car vs. solvent | 88.9 | 77.8 | 72.2 | 66.7 | 72.2 | 66.7 | | 74.1 | 3.4 | 59.4 |
| Individual pre-TUR urine mixture ($U_i$) vs. post-TUR $U_m$ or pre-TUR $U_m$ vs. individual post-TUR $U_i$ | | | | | | | | | | |
| $10^{-6}$ pre**- vs. post*-N :$U_m$ | 66.7 | 77.8 | 61.1 | 72.2 | 66.7 | 72.2 | 66.7 | 69.0 | 2.0 | 58.7 |
| $10^{-6}$ pre*-N6 :$U_i$ vs. post*-N :$U_m$ | 50.0 | 94.4 | 72.2 | 77.8 | 72.2 | 66.7 | 61.1 | 70.6 | 5.2 | 58.7 |
| $10^{-6}$ pre†-N8 :$U_i$ vs. post*-N :$U_m$ | 55.6 | 77.8 | 72.2 | 83.3 | 66.7 | 72.2 | 55.6 | 69.0 | 4.0 | 58.7 |
| $10^{-6}$ pre-N10 :$U_i$ vs. post*-N :$U_m$ | 55.6 | 83.3 | 72.2 | 77.8 | 72.2 | 61.1 | 55.6 | 68.3 | 4.1 | 58.7 |
| $10^{-6}$ pre‡-N5 :$U_i$ vs. post*-N :$U_m$ | 66.7 | 72.2 | 83.3 | 88.9 | 72.2 | 66.7 | 66.7 | 73.8 | 3.4 | 58.7 |
| $10^{-6}$ pre**- vs. post*-N :$U_m$ | 61.1 | 77.8 | 72.2 | 77.8 | 61.1 | 61.1 | 66.7 | 68.3 | 2.9 | 58.7 |
| $10^{-6}$ pre**-N :$U_m$ vs. post-N6 :$U_i$ | 66.7 | 83.3 | 77.8 | 77.8 | 72.2 | 61.1 | 66.7 | 72.2 | 3.0 | 58.7 |
| $10^{-6}$ pre**- vs. post*-N :$U_m$ | | 88.9 | 66.7 | 88.9 | 77.8 | 72.2 | | 78.9 | 4.4 | 60.3 |
| $10^{-6}$ pre**-N :$U_m$ vs. post-N8 :$U_i$ | 55.6 | 77.8 | 72.2 | 83.3 | 61.1 | 72.2 | 61.1 | 69.0 | 3.8 | 58.7 |
| $10^{-6}$ pre**-N :$U_m$ vs. post-N10 :$U_i$ | 55.6 | 83.3 | 88.9 | 83.3 | 66.7 | 72.2 | 66.7 | 73.8 | 4.5 | 58.7 |
| $10^{-6}$ pre**- vs. post*-N :$U_m$ | 66.7 | 88.9 | 94.4 | 88.9 | 72.2 | 55.6 | 55.6 | 74.6 | 6.2 | 58.7 |
| $10^{-6}$ pre**-N:Um vs. post†-N5:Ui | 55.6 | 83.3 | 55.6 | 66.7 | 83.3 | 66.7 | | 68.5 | 5.1 | 59.4 |
| $10^{-6}$ pre**- vs. post*-N :$U_m$ | 66.7 | 77.8 | 66.7 | 72.2 | 77.8 | 66.7 | 66.7 | 70.6 | 2.0 | 58.7 |
| $10^{-6}$ post-N6 :$U_i$ vs. post*-N :$U_m$ | 61.1 | 100.0 | 66.7 | 94.4 | 94.4 | 61.1 | 61.1 | 77.0 | 6.9 | 58.7 |
| $10^{-6}$ pre**- vs. post*-N :$U_m$ | 72.2 | 94.4 | 77.8 | 88.9 | 77.8 | 66.7 | 72.2 | 78.6 | 3.7 | 58.7 |
| $10^{-6}$ post-N8 :$U_i$ vs. post*-N :$U_m$ | 61.1 | 72.2 | 61.1 | 77.8 | 66.7 | 55.6 | 61.1 | 65.1 | 2.9 | 58.7 |
| $10^{-6}$ pre**- vs. post*-N :$U_m$ | 61.1 | 72.2 | 66.7 | 94.4 | 72.2 | 66.7 | 55.6 | 69.8 | 4.7 | 58.7 |
| $10^{-6}$ post-N10 :$U_i$ vs. post*-N :$U_m$ | 66.7 | 61.1 | 61.1 | 72.2 | 61.1 | 72.2 | 55.6 | 64.3 | 2.4 | 58.7 |
| $10^{-6}$ pre**- vs. post*-N :$U_m$ | 55.6 | 55.6 | 50.0 | 55.6 | 55.6 | 55.6 | 50.0 | 54.0 | 1.0 | 58.7 |
| $10^{-6}$ post†-N5 :$U_i$ vs. post*-N :$U_m$ | 50.0 | 50.0 | 50.0 | 50.0 | 50.0 | 50.0 | 50.0 | 50.0 | 0.0 | 58.7 |
| Post assays and re-training | | | | | | | | | | |
| $10^{-1}$ pre**- vs. post*-N :$U_m$ | 55.6 | 88.9 | 66.7 | 72.2 | 61.1 | 61.1 | 77.8 | 67.6 | 4.8 | 58.7 |
| $10^{-1}$ pre**- vs. post*-N :$U_m$ | 55.6 | 55.6 | 55.6 | 61.1 | 61.1 | 55.6 | 55.6 | 57.4 | 1.2 | 58.7 |
| $10^{-5}$ (-)car vs. solvent | 61.1 | 61.1 | 61.1 | 61.1 | 61.1 | 61.1 | 66.7 | 61.1 | 0.0 | 58.7 |
| $10^{-5}$ (-)car vs. solvent | 55.6 | 77.8 | 61.1 | 61.1 | 72.2 | 55.6 | 61.1 | 63.9 | 3.7 | 58.7 |
| $10^{-3}$ (-)car vs. solvent | 66.7 | 72.2 | 55.6 | 66.7 | 72.2 | 66.7 | 77.8 | 66.7 | 2.5 | 58.7 |
| $10^{-3}$ (-)car vs. solvent | 66.7 | 88.9 | 66.7 | 77.8 | 72.2 | 66.7 | 83.3 | 73.1 | 3.6 | 58.7 |
| $10^{-3}$ (-)car vs. solvent | 88.9 | 66.7 | 77.8 | 72.2 | 66.7 | 83.3 | 77.8 | 75.9 | 3.7 | 58.7 |
| Re-post assays | | | | | | | | | | |
| $10^{-1}$ pre**- vs. post*-N :$U_m$ | 66.7 | 83.3 | 72.2 | 61.1 | 66.7 | 61.1 | 61.1 | 67.5 | 3.1 | 58.7 |
| $10^{-2}$ pre**- vs. post*-N :$U_m$ | 55.6 | 72.2 | 61.1 | 72.2 | 83.3 | 61.1 | 66.7 | 67.5 | 3.5 | 58.7 |
| $10^{-6}$ pre**- vs. post*-N :$U_m$ | 50.0 | 55.6 | 38.9 | 44.4 | 55.6 | 55.6 | | 50.0 | 2.9 | 59.4 |

Extra-dilution rates for equi-occult blood urine samples were 1/6* v/v, 1/13** v/v, 1/15† v/v, and 1/75‡ v/v. SE, standard error.

**Table ST2.** %Correct of WT and ΔD mice for serial 10- or 100-fold diluted equi-occult blood urine samples in a Y-maze (continued).

| Odour pairs | %Correct of individual ΔD mice (%) | | | | | | avg. of 6 ΔD mice | SE | %Correct for $P$ = 0.05 |
|---|---|---|---|---|---|---|---|---|---|
| | *ΔD1* | *ΔD2* | *ΔD3* | *ΔD4* | *ΔD5* | *ΔD6* | | | |
| Pre**- vs. post*-TUR urine mixture ($U_m$), equi-occult blood | | | | | | | | | |
| $10^{-5}$ (-)car vs. solvent | 66.7 | 72.2 | 72.2 | 77.8 | 66.7 | 72.2 | 71.3 | 1.7 | 59.4 |
| $10^{-1}$ pre**- vs. post*-*N* :$U_m$ | 66.7 | 66.7 | 61.1 | 66.7 | 72.2 | 66.7 | 66.7 | 1.4 | 59.4 |
| $10^{-2}$ pre**- vs. post*-*N* :$U_m$ | 55.6 | 55.6 | 61.1 | 55.6 | 61.1 | 61.1 | 58.3 | 1.2 | 59.4 |
| $10^{-3}$ pre**- vs. post*-*N* :$U_m$ | 77.8 | 50.0 | 50.0 | 61.1 | 61.1 | 61.1 | 60.2 | 4.2 | 59.4 |
| $10^{-4}$ pre**- vs. post*-*N* :$U_m$ | 66.7 | 55.6 | 50.0 | 38.9 | 50.0 | 50.0 | 51.9 | 3.7 | 59.4 |
| Post assays | | | | | | | | | |
| $10^{-2}$ pre**- vs. post*-*N* :$U_m$ | 72.2 | 83.3 | 72.2 | 72.2 | 72.2 | 61.1 | 72.2 | 2.9 | 59.4 |
| $10^{-2}$ pre**- vs. pre**-*N* :$U_m$ | 50.0 | 55.6 | 50.0 | 50.0 | 55.6 | 33.3 | 49.1 | 3.3 | 59.4 |
| Healthy volunteer 1st–3rd $U_m$ (*H1–3*) vs. 4th–6th $U_m$ (*H4–6*) | | | | | | | | | |
| *H1–3* vs. *H4–6* $U_m$ | 61.1 | 55.6 | 50.0 | 66.7 | 55.6 | 55.6 | 57.4 | 2.3 | 59.4 |
| $10^{-1}$ *H1–3* vs. *H4–6* $U_m$ | 50.0 | 50.0 | 44.4 | 61.1 | 50.0 | 61.1 | 52.8 | 2.8 | 59.4 |
| $10^{-2}$ *H1–3* vs. *H4–6* $U_m$ | 50.0 | 44.4 | 44.4 | 55.6 | 55.6 | 44.4 | 49.1 | 2.2 | 59.4 |
| $10^{-3}$ *H1–3* vs. *H4–6* $U_m$ | 44.4 | 61.1 | 38.9 | 44.4 | 44.4 | 50.0 | 47.2 | 3.1 | 59.4 |
| $10^{-4}$ *H1–3* vs. *H4–6* $U_m$ | 33.3 | 44.4 | 55.6 | 72.2 | 38.9 | 33.3 | 46.3 | 6.2 | 59.4 |
| Post assays | | | | | | | | | |
| $10^{-5}$ (-)car vs. solvent | 72.2 | 61.1 | 72.2 | 66.7 | 72.2 | 55.6 | 66.7 | 2.9 | 59.4 |

Extra-dilution rates for equi-occult blood urine samples were 1/6* v/v and 1/13** v/v. (-)car, (R)-(-)-carvone (w/w); solvent, di(propylene) glycol. SE, standard error.

**Table ST3.** Blood, proteins, glucose testing in *N*-series patient urine samples using urine test strips.

| Parameters and diluted urine samples | Pre-TUR urine samples | | | | | | | Post-TUR urine samples | | | | | | |
|---|---|---|---|---|---|---|---|---|---|---|---|---|---|---|
| | *N5*:$U_i$ | *N6*:$U_i$ | *N7*:$U_i$ | *N8*:$U_i$ | *N9*:$U_i$ | *N10*:$U_i$ | *N*:$U_m$ | *N5*:$U_i$ | *N6*:$U_i$ | *N7*:$U_i$ | *N8*:$U_i$ | *N9*:$U_i$ | *N10*:$U_i$ | *N*:$U_m$ |
| Cancer | blad. | blad. | blad. | blad. | blad. | blad. | blad. | | | | | | | |
| Stage | I | I | I | I | I | I | I | | | | | | | |
| Grade (H: high; L: low) | L | L | L | H | H | H | 3H+2L | | | | | | | |
| Age | 76 | 85 | 78 | 81 | 69 | 91 | 69–91 | | | | | | | |
| Gender | ♀ | ♂ | ♂ | ♂ | ♂ | ♂ | ♂ | | | | | | | |
| Sampling (5 days) | (-29)–(-1) | (-53)–(-1) | (-35)–(-1) | (-42)–(-1) | (-49)–(-1) | (-21)–(-1) | (-53)–(-1) | 9–38 | 14–20 | 8–120 | 10–122 | 11–123 | 15–127 | 8–127 |
| Haemoglobin (Hb, blood) (mg/dℓ) | | | | | | | | | | | | | | |
| stock $U_i/U_m$ (1st) | 0.75 | 0.06 | 0.00 | 0.15 | 0.10 | 0.00 | 0.15 | 0.15 | 0.00 | 0.03 | 0.00 | 0.06 | 0.00 | 0.06 |
| stock $U_i/U_m$ (2nd) | | | | | | | 0.11 | | | | | | | 0.06 |
| average for $U_i/U_m$ | 0.75 | 0.06 | 0.00 | 0.15 | 0.10 | 0.00 | 0.13 | 0.15 | 0.00 | 0.03 | 0.00 | 0.06 | 0.00 | 0.06 |
| $10^{-1}$-diluted $U_i/U_m$ | 0.11 | 0.00 | 0.00 | 0.00 | 0.00 | 0.00 | 0.00 | 0.06 | 0.00 | 0.00 | 0.00 | 0.00 | 0.00 | 0.00 |
| $10^{-2}$-diluted $U_i/U_m$ | 0.00 | 0.00 | 0.00 | 0.00 | 0.00 | 0.00 | 0.00 | 0.00 | 0.00 | 0.00 | 0.00 | 0.00 | 0.00 | 0.00 |
| $10^{-3}$-diluted $U_i/U_m$ | 0.00 | 0.00 | 0.00 | 0.00 | 0.00 | 0.00 | 0.00 | 0.00 | 0.00 | 0.00 | 0.00 | 0.00 | 0.00 | 0.00 |
| Dilution for equi-occult blood of 0.01 mg/dℓ or less Hb | | | | | | | | | | | | | | |
| Extra-dilution rate | 75 | 6 | 1 | 15 | 10 | 1 | 13 | 15 | 1 | 3 | 1 | 6 | 1 | 6 |
| Protein (mg/dℓ) | | | | | | | | | | | | | | |
| stock $U_i/U_m$ (1st) | 0.0 | 0.0 | 0.0 | 15.0 | 0.0 | 0.0 | 0.0 | 22.5 | 15.0 | 20.0 | 15.0 | 20.0 | 0.0 | 15.0 |
| stock $U_i/U_m$ (2nd) | | | | | | | 15.0 | | | | | | | 0.0 |
| average for $U_i/U_m$ | 0.0 | 0.0 | 0.0 | 15.0 | 0.0 | 0.0 | 7.5 | 22.5 | 15.0 | 20.0 | 15.0 | 20.0 | 0.0 | 7.5 |
| $10^{-1}$-diluted $U_i/U_m$ | 0.0 | 0.0 | 0.0 | 0.0 | 0.0 | 0.0 | 0.0 | 15.0 | 0.0 | 0.0 | 0.0 | 0.0 | 0.0 | 0.0 |
| $10^{-2}$-diluted $U_i/U_m$ | 0.0 | 0.0 | 0.0 | 0.0 | 0.0 | 0.0 | 0.0 | 0.0 | 0.0 | 0.0 | 0.0 | 0.0 | 0.0 | 0.0 |
| $10^{-3}$-diluted $U_i/U_m$ | 0.0 | 0.0 | 0.0 | 0.0 | 0.0 | 0.0 | 0.0 | 0.0 | 0.0 | 0.0 | 0.0 | 0.0 | 0.0 | 0.0 |
| Glucose (mg/dℓ) | | | | | | | | | | | | | | |
| stock $U_i/U_m$ (1st) | 0 | 0 | 0 | 0 | >2000 | 0 | 50 | 500 | 0 | 100 | 0 | 0 | 0 | 0 |
| stock $U_i/U_m$ (2nd) | | | | | | | 50 | | | | | | | 0 |
| average for $U_i/U_m$ | 0 | 0 | 0 | 0 | >2000 | 0 | 50 | 500 | 0 | 100 | 0 | 0 | 0 | 0 |
| $10^{-1}$-diluted $U_i/U_m$ | 0 | 0 | 0 | 0 | 500 | 0 | 0 | 50 | 0 | 0 | 0 | 0 | 0 | 0 |
| $10^{-2}$-diluted $U_i/U_m$ | 0 | 0 | 0 | 0 | 50 | 0 | 0 | 0 | 0 | 0 | 0 | 0 | 0 | 0 |
| $10^{-3}$-diluted $U_i/U_m$ | 0 | 0 | 0 | 0 | 0 | 0 | 0 | 0 | 0 | 0 | 0 | 0 | 0 | 0 |

$U_i$, individual patient urine mixture ($U_i$) of equal volumes of five urine samples from each patient. *N*:$U_m$, *N*-series urine mixture ($U_m$) of equal volumes of 25 urine samples from five patient *N6–N10* on five different days for each pre- and post-transurethral resection (post-TUR) after antibiotic exposure. Abbreviation: blad., bladder. Ranges of patient $1^{st}$–$5^{th}$ sampling days are shown, when day 0 is the ablative operation day of the patient.

**Table ST4.** Blood, proteins, glucose testing in *K* -, and *A* -series patient and healthy volunteer urine samples using urine test strips.

| Parameters and diluted urine samples | Pre-TUR urine samples | | | | | | Post-TUR urine samples | | | | | | Healthy $U_m$ | |
|---|---|---|---|---|---|---|---|---|---|---|---|---|---|---|
| | *K3* :$U_i$ | *K4* :$U_i$ | *K5* :$U_i$ | *K* :$U_m$ | *A2* :$U_i$ | *A3* :$U_i$ | *K3* :$U_i$ | *K4* :$U_i$ | *K5* :$U_i$ | *K* :$U_m$ | *A2* :$U_i$ | *A3* :$U_i$ | *H1-3* : $U_m$ | *H4-6* : $U_m$ |
| Cancer | blad. | blad. | blad. | blad. | blad. | blad. | | | | | | | none | |
| Stage | I | I | I | I | I | I | | | | | | | - | |
| Grade (H: high; L: low) | H | L | H | 4H+1L | H | H | | | | | | | - | |
| Age | 80 | 69 | 76 | 69–96 | 84 | 71 | | | | | | | 52–75 | |
| Gender | ♂ | ♂ | ♂ | ♂ | ♂ | ♂ | | | | | | | ♂ | |
| Sampling (5 days) | (-42)–(-1) | (-60)–(-1) | (-35)–(-1) | (-60)–(-1) | (-26)–(-1) | (-31)–(-1) | 1–5 | 1–5 | 1–5 | 1–5*, 8–123** | 9–38 | 14–20 | (-17)–(-1)‡ | 0–11¶ |
| Haemoglobin (Hb, blood) (mg/dℓ) | | | | | | | | | | | | | | |
| stock $U_i$/$U_m$ (1st) | 0.15 | 0.00 | 0.00 | 0.06 | 0.09 | 0.00 | >0.75 | 0.75 | 0.00 | >0.75 | 0.13 | 0.00 | 0.00 | 0.00 |
| stock $U_i$/$U_m$ (2nd) | | | | 0.06 | | | | | | >0.75 | | | | |
| average for $U_i$/$U_m$ | 0.15 | 0.00 | 0.00 | 0.06 | 0.09 | 0.00 | >0.75 | 0.75 | 0.00 | >0.75 | 0.13 | 0.00 | 0.00 | 0.00 |
| $10^{-1}$-diluted $U_i$/$U_m$ | 0.00 | 0.00 | 0.00 | 0.00 | 0.00 | 0.00 | 0.15 | 0.11 | 0.00 | >0.15 | 0.00 | 0.00 | 0.00 | 0.00 |
| $10^{-2}$-diluted $U_i$/$U_m$ | 0.00 | 0.00 | 0.00 | 0.00 | 0.00 | 0.00 | 0.03 | 0.03 | 0.00 | 0.06 | 0.00 | 0.00 | 0.00 | 0.00 |
| $10^{-3}$-diluted $U_i$/$U_m$ | 0.00 | 0.00 | 0.00 | 0.00 | 0.00 | 0.00 | 0.00 | 0.00 | 0.00 | 0.00 | 0.00 | 0.00 | | |
| Dilution for equi-occult blood of 0.01 mg/dℓ or less Hb | | | | | | | | | | | | | | |
| Extra-dilution rate | 15 | 1 | 1 | 6 | 9 | 1 | 130† | 75 | 1 | – | 13 | 1 | 1 | 1 |
| Protein (mg/dℓ) | | | | | | | | | | | | | | |
| stock $U_i$/$U_m$ (1st) | 15.0 | 0.0 | 0.0 | 0.0 | 8.0 | 0.0 | 15.0 | 0.0 | 15.0 | 15.0 | 15.0 | 15.0 | 0.0 | 0.0 |
| stock $U_i$/$U_m$ (2nd) | | | | 0.0 | | | | | | 30.0 | | | | |
| average for $U_i$/$U_m$ | 15.0 | 0.0 | 0.0 | 0.0 | 8.0 | 0.0 | 15.0 | 0.0 | 15.0 | 22.5 | 15.0 | 15.0 | 0.0 | 0.0 |
| $10^{-1}$-diluted $U_i$/$U_m$ | 0.0 | 0.0 | 0.0 | 0.0 | 0.0 | 0.0 | 0.0 | 0.0 | 0.0 | 0.0 | 0.0 | 0.0 | 0.0 | 0.0 |
| $10^{-2}$-diluted $U_i$/$U_m$ | 0.0 | 0.0 | 0.0 | 0.0 | 0.0 | 0.0 | 0.0 | 0.0 | 0.0 | 0.0 | 0.0 | 0.0 | 0.0 | 0.0 |
| $10^{-3}$-diluted $U_i$/$U_m$ | 0.0 | 0.0 | 0.0 | 0.0 | 0.0 | 0.0 | 0.0 | 0.0 | 0.0 | 0.0 | 0.0 | 0.0 | | |
| Glucose (mg/dℓ) | | | | | | | | | | | | | | |
| stock $U_i$/$U_m$ (1st) | 500 | 0 | 0 | 0 | 50 | 0 | 500 | 0 | 100 | 300 | 50 | 0 | 2000 | 2000 |
| stock $U_i$/$U_m$ (2nd) | | | | 50 | | | | | | 500 | | | | |
| average for $U_i$/$U_m$ | 500 | 0 | 0 | 25 | 50 | 0 | 500 | 0 | 100 | 400 | 50 | 0 | 2000 | 2000 |
| $10^{-1}$-diluted $U_i$/$U_m$ | 0 | 0 | 0 | 0 | 0 | 0 | 300 | 0 | 0 | 100 | 0 | 0 | 500 | 500 |
| $10^{-2}$-diluted $U_i$/$U_m$ | 0 | 0 | 0 | 0 | 0 | 0 | 100 | 0 | 0 | 25 | 0 | 0 | 50 | 50 |
| $10^{-3}$-diluted $U_i$/$U_m$ | 0 | 0 | 0 | 0 | 0 | 0 | 0 | 0 | 0 | 0 | 0 | 0 | | |

$U_i$, individual patient urine mixture ($U_i$) of equal volumes of five urine samples from each patient. *K* :$U_m$, *K* -series urine mixture ($U_m$) of equal volumes of 25 urine samples from five patient *K3–K5* , *A1* and *T2* on five different days for each pre-TUR and antibiotic-exposure post-TUR except for a patient (four patients* + one patient**). Abbreviation: blad., bladder. Extra-dilution rates of urine samples were determined by using linear regression of Hb = (0.15, 0.03)† for diluted urine = ($10^{-1}$, $10^{-2}$). *H1–3* , an equal-volume mixture of 18 urine samples from six healthy volunteer on three ($n$ = 5) or two ($n$ = 1, including one-day AM and PM sampling) different days; *H4–6* , an equal-volume mixture of 18 urine samples from the identical six healthy volunteers on three different days. Ranges of patient 1st–5th, healthy volunteer 1st–3rd‡ and 4th–6th¶ sampling days are shown, when day 0 is the ablative operation day of the patient or the fourth sampling day of the healthy volunteer.

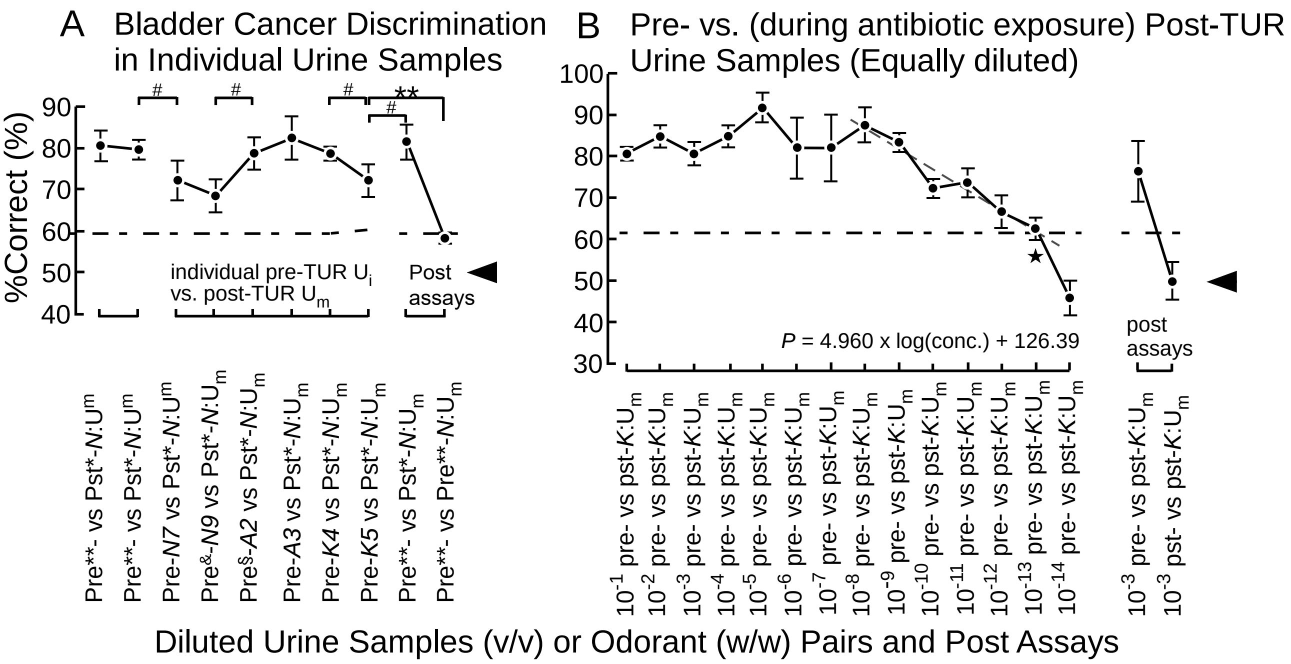


**Fig. S2.** Urine odour discrimination performance of wild-type (WT) mice for individual patient urine mixtures ($U_i$) and antibiotic drug metabolite-containing urine mixtures ($U_m$) in the Y-maze. (*A*), Odour discrimination between equi-occult blood individual patient pre-TUR $U_i$ vs. post-TUR $U_m$ after re-training with the equi-occult blood pre- vs. post-TUR $U_m$. Extra-dilution rates for equi-occult blood $U_m$s were 1/6* v/v, 1/9§ v/v, 1/10& v/v, and 1/13** v/v. *P* value of paired difference in %Correct is indicated by #($P \geq 0.05$) and **($0.001 \leq P < 0.01$).(*B*), Odour discrimination between equally-diluted pre- vs. (during antibiotic exposure) post-TUR $U_m$ of other five patients with bladder cancer. Post assays: $10^{-3}$ pre- vs. post-TUR $U_m$ and identical $U_m$ pair: $10^{-3}$ post- vs. post-TUR $U_m$. Tasks performed at thresholds are marked by the star. A linear regression model is shown (grey dashed line). Chain lines indicate %Correct significantly above chance performance, $P$ = 0.05 for 72 to 108 trials, 18 trials/mouse). Black arrowheads indicate chance levels (50%).

**Table ST5. %**Correct of wild-type (WT) mice for serial diluted urine samples and urine discrimination in a Y-maze.

| Odour pairs | %Correct of individual WT mice (%) | | | | | | avg. of 6 WT mice | SE | *P* value | %Correct for *P* = 0.05 |
|---|---|---|---|---|---|---|---|---|---|---|
| | *wt8* | *wt9* | *wt10* | *wt11* | *wt12* | *wt13* | | | | |
| Pre- vs. post-TUR urine mixture ($U_m$) | | | | | | | | | | |
| $10^{-9}$ (-)car vs. (+)car | 72.2 | 72.2 | 72.2 | 77.8 | 77.8 | 66.7 | 73.1 | 1.7 | | 59.4 |
| $10^{-1}$ pre- vs. post-*N* :$U_m$ | 77.8 | 83.3 | 83.3 | 72.2 | 72.2 | 77.8 | 77.8 | 2.0 | | 59.4 |
| $10^{-2}$ pre- vs. post-*N* :$U_m$ | 77.8 | 72.2 | 72.2 | 94.4 | 83.3 | 77.8 | 79.6 | 3.4 | | 59.4 |
| $10^{-3}$ pre- vs. post-*N* :$U_m$ | 72.2 | 72.2 | 72.2 | 77.8 | 72.2 | 72.2 | 73.1 | 0.9 | | 59.4 |
| $10^{-4}$ pre- vs. post-*N* :$U_m$ | 66.7 | 83.3 | 72.2 | 72.2 | 77.8 | 83.3 | 75.9 | 2.7 | | 59.4 |
| $10^{-5}$ pre- vs. post-*N* :$U_m$ | 72.2 | 88.9 | 88.9 | 72.2 | 72.2 | 77.8 | 78.7 | 3.3 | | 59.4 |
| $10^{-6}$ pre- vs. post-*N* :$U_m$ | 83.3 | 94.4 | 83.3 | 66.7 | 94.4 | 94.4 | 86.1 | 4.5 | | 59.4 |
| $10^{-8}$ pre- vs. post-*N* :$U_m$ | 72.2 | 72.2 | 72.2 | 72.2 | 61.1 | 77.8 | 71.3 | 2.2 | | 59.4 |
| $10^{-10}$ pre- vs. post-*N* :$U_m$ | 66.7 | 44.4 | 66.7 | 61.1 | 55.6 | 66.7 | 60.2 | 3.6 | | 59.4 |
| $10^{-12}$ pre- vs. post-*N* :$U_m$ | 61.1 | 50.0 | 61.1 | 55.6 | 55.6 | 55.6 | 56.5 | 1.7 | | 59.4 |
| Post assays | | | | | | | | | | |
| $10^{-6}$ pre- vs. post-*N* :$U_m$ | 77.8 | 72.2 | 72.2 | 72.2 | 77.8 | 77.8 | 75.0 | 1.2 | | 59.4 |
| $10^{-6}$ pre- vs. pre-*N* :$U_m$ | 61.1 | 44.4 | 55.6 | 55.6 | 61.1 | 50.0 | 54.6 | 2.7 | | 59.4 |
| Individual pre-TUR urine ($U_i$) vs. post-TUR $U_m$ after training with equally-diluted $U_m$ pair | | | | | | | | | | |
| $10^{-6}$ pre- vs. post-*N* :$U_m$ | 77.8 | 77.8 | 83.3 | 72.2 | 77.8 | 88.9 | 79.6 | 2.3 | 6.E-04 | 59.4 |
| $10^{-6}$ pre∗-*N6* :$U_i$ vs. post*-*N* :$U_m$ | 66.7 | 61.1 | 61.1 | 66.7 | 72.2 | 61.1 | 64.8 | 1.9 | | 59.4 |
| $10^{-6}$ pre- vs. post-*N* :$U_m$ | 66.7 | 55.6 | 61.1 | 55.6 | 55.6 | 72.2 | 61.1 | 2.9 | 1.E-04 | 59.4 |
| $10^{-6}$ pre- vs. post-*N* :$U_m$ | 88.9 | 83.3 | 77.8 | 77.8 | 94.4 | 83.3 | 84.3 | 2.7 | 0.070 | 59.4 |
| $10^{-6}$ pre†-*N8* :$U_i$ vs. post*-*N* :$U_m$ | 72.2 | 83.3 | 88.9 | 66.7 | 72.2 | 66.7 | 75.0 | 3.7 | 0.018 | 59.4 |
| $10^{-6}$ pre- vs. post-*N* :$U_m$ | 66.7 | 72.2 | 61.1 | 55.6 | 55.6 | 61.1 | 62.0 | 2.7 | | 59.4 |
| $10^{-6}$ pre- vs. post-*N* :$U_m$ | 61.1 | 66.7 | 55.6 | 61.1 | 66.7 | 66.7 | 63.0 | 1.9 | 0.002 | 59.4 |
| $10^{-6}$ pre-*N10* :$U_i$ vs. post*-*N* :$U_m$ | 66.7 | 72.2 | 77.8 | 77.8 | 72.2 | 77.8 | 74.1 | 1.9 | 0.028 | 59.4 |
| $10^{-6}$ pre- vs. post-*N* :$U_m$ | 66.7 | 77.8 | 55.6 | 61.1 | 66.7 | 61.1 | 64.8 | 3.1 | 0.003‡ | 59.4 |
| Individual pre-TUR urine ($U_i$) or $U_m$ vs. post-TUR $U_m$ after re-training with the equi-occult blood $U_m$ pair | | | | | | | | | | |
| $10^{-6}$ pre**- vs. post*-*N* :$U_m$ | 66.7 | 61.1 | 55.6 | 66.7 | 55.6 | 77.8 | 63.9 | 3.4 | | 59.4 |
| $10^{-3}$ pre**- vs. post*-*N* :$U_m$ | 66.7 | 61.1 | 72.2 | 66.7 | 61.1 | 66.7 | 65.7 | 1.7 | 0.044 | 59.4 |
| $10^{-3}$ pre**- vs. post*-*N* :$U_m$ | 66.7 | 72.2 | 72.2 | 77.8 | 66.7 | 72.2 | 71.3 | 1.7 | 0.156 | 59.4 |
| $10^{-6}$ pre**- vs. post*-*N* :$U_m$ | 61.1 | 72.2 | 66.7 | 72.2 | 66.7 | 66.7 | 67.6 | 1.7 | 0.014 | 59.4 |
| $10^{-6}$ pre**- vs. post*-*N* :$U_m$ | 83.3 | 72.2 | 72.2 | 83.3 | 72.2 | 94.4 | 79.6 | 3.7 | 0.511 | 59.4 |
| $10^{-6}$ pre†-*N8* :$U_i$ vs. post*-*N* :$U_m$ | 72.2 | 83.3 | 72.2 | 61.1 | 88.9 | 77.8 | 75.9 | 4.0 | 0.166 | 59.4 |
| $10^{-6}$ pre**- vs. post*-*N* :$U_m$ | 66.7 | 61.1 | 55.6 | 55.6 | 83.3 | 77.8 | 66.7 | 4.8 | 0.013 | 59.4 |
| $10^{-6}$ pre**- vs. post*-*N* :$U_m$ | 77.8 | 88.9 | 88.9 | 83.3 | 77.8 | 77.8 | 82.4 | 2.2 | 0.283 | 59.4 |
| $10^{-6}$ pre†-*K3* :$U_i$ vs. post*-*N* :$U_m$ | 77.8 | 94.4 | 77.8 | 94.4 | 83.3 | 94.4 | 87.0 | 3.4 | 0.341 | 59.4 |
| $10^{-6}$ pre**- vs. post*-*N* :$U_m$ | 88.9 | 83.3 | 83.3 | 77.8 | 83.3 | 83.3 | 83.3 | 1.4 | 3.E-04 | 59.4 |
| $10^{-6}$ pre-*K4* :$U_i$ vs. post*-*N* :$U_m$ | 72.2 | 77.8 | 72.2 | 66.7 | 72.2 | 72.2 | 72.2 | 1.4 | 0.473 | 59.4 |
| $10^{-6}$ pre**- vs. post*-*N* :$U_m$ | 77.8 | 72.2 | 66.7 | 77.8 | 66.7 | 88.9 | 75.0 | 3.4 | 0.414 | 59.4 |
| $10^{-6}$ pre**- vs. post*-*N* :$U_m$ | 77.8 | 77.8 | 72.2 | 83.3 | 88.9 | 72.2 | 78.7 | 2.7 | 0.040 | 59.4 |
| $10^{-6}$ pre-*K5* :$U_i$ vs. post*-*N* :$U_m$※ | 66.7 | 77.8 | 55.6 | 77.8 | 66.7 | 66.7 | 68.5 | 3.4 | | 59.4 |
| Post assays | | | | | | | | | | |
| $10^{-6}$ pre**- vs. post*-*N* :$U_m$ | 77.8 | 83.3 | 77.8 | 72.2 | 77.8 | 77.8 | 77.8 | 1.4 | 2.E-06 | 59.4 |
| $10^{-6}$ pre**- vs. pre**-*N* :$U_m$ | 50.0 | 55.6 | 50.0 | 38.9 | 55.6 | 50.0 | 50.0 | 2.5 | | 59.4 |
| Individual pre-TUR urine ($U_i$) or Um vs. post-TUR $U_m$ after re-training with the equi-occult blood $U_m$ pair | | | | | | | | | | |
| $10^{-6}$ pre**- vs. post*-*N* :$U_m$ | 77.8 | 94.4 | 77.8 | 72.2 | 72.2 | 88.9 | 80.6 | 3.7 | | 59.4 |
| $10^{-6}$ pre**- vs. post*-*N* :$U_m$ | 77.8 | 77.8 | 83.3 | 72.2 | 88.9 | 77.8 | 79.6 | 2.3 | 0.193 | 59.4 |
| $10^{-6}$ pre-*N7* :$U_i$ vs. post*-*N* :$U_m$ | 61.1 | 66.7 | 72.2 | 72.2 | 66.7 | 94.4 | 72.2 | 4.8 | | 59.4 |
| $10^{-6}$ pre†-*N9* :$U_i$ vs. post*-*N* :$U_m$ | 61.1 | 66.7 | 77.8 | 61.1 | 61.1 | 83.3 | 68.5 | 4.0 | 0.097 | 59.4 |
| $10^{-6}$ pre**-*A2* :$U_i$ vs. post*-*N* :$U_m$ | 61.1 | 88.9 | 83.3 | 77.8 | 77.8 | 83.3 | 78.7 | 3.9 | | 59.4 |
| $10^{-6}$ pre-*A3* :$U_i$ vs. post*-*N* :$U_m$ | 61.1 | 83.3 | 83.3 | 100.0 | 77.8 | 88.9 | 82.4 | 5.3 | | 59.4 |
| $10^{-6}$ pre-*K4* :$U_i$ vs. post*-*N* :$U_m$ | 72.2 | 77.8 | 77.8 | 83.3 | 77.8 | 83.3 | 78.7 | 1.7 | 0.141 | 59.4 |
| $10^{-6}$ pre-*K5* :$U_i$ vs. post*-*N* :$U_m$※ | 38.9# | 77.8 | 61.1 | 66.7 | 72.2 | 83.3 | 72.2 | 3.9 | 0.149 | 60.3 |
| Post assays | | | | | | | | | | |
| $10^{-6}$ pre**- vs. post*-*N* :$U_m$ | 83.3 | 100.0 | 72.2 | 77.8 | 72.2 | 83.3 | 81.5 | 4.2 | | 59.4 |
| $10^{-6}$ pre**- vs. pre**-*N* :$U_m$ | 55.6 | 55.6 | 61.1 | 61.1 | 55.6 | 61.1 | 58.3 | 1.2 | 0.005¶ | 59.4 |

Extra-dilution rates for equi-occult blood urine samples were 1/6* v/v, 1/9§ v/v, 1/10& v/v, 1/13** v/v, and 1/15† v/v. (+)-car, (*S* )-(+)-carvone (w/w). An outlier of the %Correct = 38.9# (statistic T = 2.384 > $T_0$ = 2.285, *P* = 0.05, one-way) among 12 data※ for *K5* : $U_i$ was excluded from analysis. ‡*P* value is for the *N* :$U_m$ pair of the first and last %Correct, and ¶*P* value is for the pair of the *K5* $U_i$ and last identical $U_m$ %Correct. SE, standard error.

**Table ST6. %**Correct of wild-type (WT) mice for re-training and individual pre- and post-TUR urine mixture ($U_i$) odor discrimination in a Y-maze.

| Odour pairs | %Correct of individual WT mice (%) | | | | | | avg. of 6 WT mice | SE | *P* value | %Correct for *P* = 0.05 |
|---|---|---|---|---|---|---|---|---|---|---|
| | *wt8* | *wt9* | *wt10* | *wt11* | *wt12* | *wt13* | | | | |
| Re-training with pre- vs. post-TUR urine mixture ($U_m$) | | | | | | | | | | |
| $10^{-2}$ pre**- vs. post*-*N* :$U_m$ | 66.7 | 77.8 | 72.2 | 77.8 | 66.7 | 94.4 | 75.9 | 4.2 | | 59.4 |
| $10^{-2}$ pre**- vs. post*-*N* :$U_m$ | 77.8 | 100.0 | 100.0 | 88.9 | 88.9 | 100.0 | 92.6 | 3.7 | | 59.4 |
| $10^{-3}$ pre**- vs. post*-*N* :$U_m$ | 77.8 | 83.3 | 77.8 | 61.1 | 72.2 | 83.3 | 75.9 | 3.4 | | 59.4 |
| $10^{-3}$ pre**- vs. post*-*N* :$U_m$ | 55.6 | 88.9 | 94.4 | 77.8 | 72.2 | 77.8 | 77.8 | 5.6 | | 59.4 |
| $10^{-3}$ pre**- vs. post*-*N* :$U_m$ | 77.8 | 94.4 | 83.3 | 83.3 | 72.2 | 83.3 | 82.4 | 3.0 | | 59.4 |
| $10^{-4}$ pre**- vs. post*-*N* :$U_m$ | 66.7 | 83.3 | 83.3 | 72.2 | 66.7 | 88.9 | 76.9 | 3.9 | | 59.4 |
| $10^{-4}$ pre**- vs. post*-*N* :$U_m$ | 77.8 | 100.0 | 77.8 | 66.7 | 72.2 | 83.3 | 79.6 | 4.7 | | 59.4 |
| $10^{-6}$ pre**- vs. post*-*N* :$U_m$ | 77.8 | 94.4 | 77.8 | 72.2 | 72.2 | 88.9 | 80.6 | 3.7 | | 59.4 |
| $10^{-6}$ pre**- vs. post*-*N* :$U_m$ | 77.8 | 77.8 | 83.3 | 72.2 | 88.9 | 77.8 | 79.6 | 2.3 | | 59.4 |
| $10^{-6}$ pre**- vs. post*-*N* :$U_m$ | 83.3 | 88.9 | 83.3 | 83.3 | 72.2 | 94.4 | 84.3 | 3.0 | | 59.4 |
| $10^{-6}$ pre**- vs. post*-*N* :$U_m$ | 83.3 | 94.4 | 83.3 | 83.3 | 77.8 | 83.3 | 84.3 | 2.2 | | 59.4 |
| Odor discrimination for individual pre- vs. post-TUR urine ($U_i$) pairs | | | | | | | | | | |
| $10^{-6}$ pre- vs. post¶-*N7* :$U_i$ | 83.3 | 94.4 | 100.0 | 77.8 | 77.8 | 88.9 | 87.0 | 3.7 | 0.162 | 59.4 |
| $10^{-6}$ pre&- vs. post*-*N9* :$U_i$ | 61.1 | 100.0 | 72.2 | 66.7 | 77.8 | 83.3 | 76.9 | 5.6 | | 59.4 |
| $10^{-6}$ pre§- vs. post**-*A2* :$U_i$ | 83.3 | 94.4 | 77.8 | 77.8 | 88.9 | 94.4 | 86.1 | 3.1 | 0.094 | 59.4 |
| $10^{-6}$ pre- vs. post-*A3* :$U_i$ | 66.7 | 83.3 | 66.7 | 88.9 | 72.2 | 83.3 | 76.9 | 3.9 | | 59.4 |
| | | | | | | | | | 0.726 | |
| Post assays | | | | | | | | | | |
| $10^{-6}$ pre**- vs. post*-*N* :$U_m$ | 77.8 | 94.4 | 72.2 | 77.8 | 77.8 | 72.2 | 78.7 | 3.3 | | 59.4 |
| $10^{-6}$ pre**- vs. pre**-*N* :$U_m$ | 44.4 | 61.1 | 55.6 | 61.1 | 61.1 | 55.6 | 56.5 | 2.7 | 0.002# | 59.4 |

Extra-dilution rates for equi-occult blood urine samples were 1/3¶ v/v, 1/6* v/v, 1/9§ v/v, 1/10& v/v, and 1/13** v/v. *P* value is for the pair of successive %Correct or the pair of *A3* :$U_i$ and identical *N* :$U_m$#. SE, standard error.

**Table ST7.** %Correct of wild-type (WT) mice for serial 10-fold diluted mixtures of urine samples collected during antibiotic exposure after transurethral resection (post-TUR).

| Odour pairs | %Correct of individual WT mice (%) | | | | avg. of 4 WT mice | SE | %Correct for *P* = 0.05 |
|---|---|---|---|---|---|---|---|
| | *wt14* | *wt15* | *wt16* | *wt17* | | | |
| Pre- vs. post-TUR urine mixture ($U_m$) | | | | | | | |
| $10^{-1}$ pre- vs. post-*K* :$U_m$ | 77.8 | 83.3 | 77.8 | 83.3 | 80.6 | 1.6 | 61.5 |
| $10^{-2}$ pre- vs. post-*K* :$U_m$ | 88.9 | 83.3 | 77.8 | 88.9 | 84.7 | 2.7 | 61.5 |
| $10^{-3}$ pre- vs. post-*K* :$U_m$ | 83.3 | 83.3 | 72.2 | 83.3 | 80.6 | 2.8 | 61.5 |
| $10^{-4}$ pre- vs. post-*K* :$U_m$ | 88.9 | 77.8 | 88.9 | 83.3 | 84.7 | 2.7 | 61.5 |
| $10^{-5}$ pre- vs. post-*K* :$U_m$ | 94.4 | 100.0 | 88.9 | 83.3 | 91.7 | 3.6 | 61.5 |
| $10^{-6}$ pre- vs. post-*K* :$U_m$ | 94.4 | 88.9 | 61.1 | 83.3 | 81.9 | 7.3 | 61.5 |
| $10^{-7}$ pre- vs. post-*K* :$U_m$ | 83.3 | 100.0 | 61.1 | 83.3 | 81.9 | 8.0 | 61.5 |
| $10^{-8}$ pre- vs. post-*K* :$U_m$ | 94.4 | 94.4 | 83.3 | 77.8 | 87.5 | 4.2 | 61.5 |
| $10^{-9}$ pre- vs. post-*K* :$U_m$ | 83.3 | 88.9 | 83.3 | 77.8 | 83.3 | 2.3 | 61.5 |
| $10^{-10}$ pre- vs. post-*K* :$U_m$ | 72.2 | 77.8 | 72.2 | 66.7 | 72.2 | 2.3 | 61.5 |
| $10^{-11}$ pre- vs. post-*K* :$U_m$ | 83.3 | 72.2 | 72.2 | 66.7 | 73.6 | 3.5 | 61.5 |
| $10^{-12}$ pre- vs. post-*K* :$U_m$ | 77.8 | 61.1 | 61.1 | 66.7 | 66.7 | 3.9 | 61.5 |
| $10^{-13}$ pre- vs. post-*K* :$U_m$ | 66.7 | 55.6 | 61.1 | 66.7 | 62.5 | 2.7 | 61.5 |
| $10^{-14}$ pre- vs. post-*K* :$U_m$ | 55.6 | 38.9 | 38.9 | 50.0 | 45.8 | 4.2 | 61.5 |
| Post assays | | | | | | | |
| $10^{-3}$ pre- vs. post-*K* :$U_m$ | 88.9 | 88.9 | 66.7 | 61.1 | 76.4 | 7.3 | 61.5 |
| $10^{-3}$ post- vs. post-*K* :$U_m$ | 50.0 | 61.1 | 50.0 | 38.9 | 50.0 | 4.5 | 61.5 |

SE, standard error.
